# Extraction, purification, structural characterization, bioactivities, and applications of *Pholiota nameko* polysaccharide: a review

**DOI:** 10.1039/d5ra08228e

**Published:** 2026-04-07

**Authors:** Jialu Sun, Jiali Zhang, Yangyi Lai, Miao Ding, Mingqi Han, Xiaoxia Kong, Zheng Li, Yifei Bian

**Affiliations:** a School of Medicine, Shandong University of Traditional Chinese Medicine Jinan 250355 China tcvmyifei@sina.com; b School of Pharmaceutical Sciences, Shandong University of Traditional Chinese Medicine Jinan 250355 China 1639651543@qq.com; c Innovation Research Institute of Traditional Chinese Medicine, Shandong University of Traditional Chinese Medicine Jinan 250355 China

## Abstract

*Pholiota nameko*, an edible mushroom, is recognized for its significant medicinal properties and broad application potential. *Pholiota nameko* polysaccharide (PNP), a major bioactive component derived from this fungus, exhibits diverse biological activities, including anti-inflammatory, antioxidant, antitumor, hypoglycemic, hypolipidemic, and immunomodulatory effects. As a natural polysaccharide, PNP also holds considerable value in the food industry, where it functions as a thickener, gelling agent, and more. This review comprehensively summarizes recent advances in the extraction, purification, structural characterization, biological activities, structure-activity relationships, and food applications of PNP. Furthermore, it provides a foundation for future research aimed at optimizing extraction and purification techniques, elucidating structure-activity relationships, exploring structural modification strategies, and developing functional food products based on PNP.

## Abstract 2

The flow chart of extraction, separation and purification of PNP.

## Graphical abstract

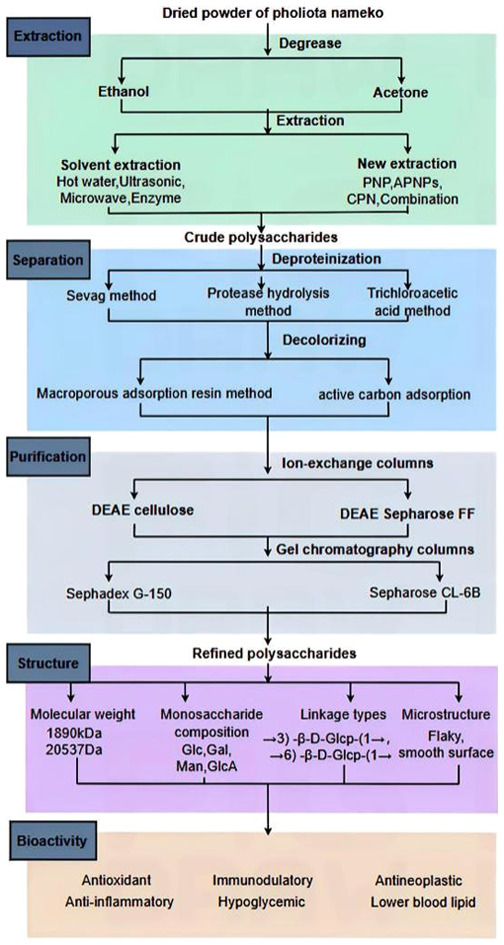

## 1. Introduction

Over the past few decades, polysaccharides derived from edible and medicinal fungi have attracted considerable scientific interest due to their diverse and potent biological activities, such as immunomodulatory, antitumor, and antioxidant effects.^1,2^ Prominent examples include lentinan from *Lentinus edodes* and a polysaccharide-peptide from *Coriolus versicolor*, which have been extensively studied and even applied in clinical settings.^3^ In addition, this growing research attention stems from the pursuit of natural, effective, and low-toxicity therapeutic agents and functional food ingredients.

**Fig. 1:**
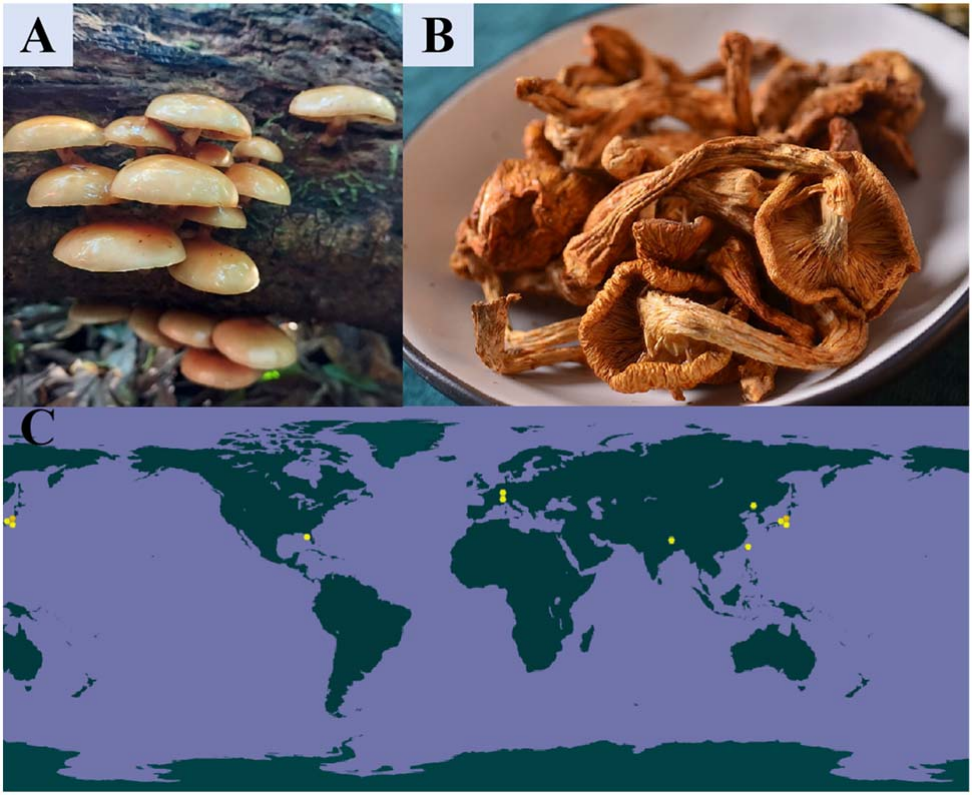
*Pholiota nameko*: (A) whole plant; (B) dried *Pholiota nameko*; (C) world distribution (Source: https://www.gbif.org).

Among the many bioactive fungi, *Pholiota nameko* stands out due to the unique slimy layer covering its fruiting bodies.^4^ Initial studies on *Pholiota nameko* date back to the mid-20th century, gradually revealing its nutritional value. In 1976, Japanese researcher Yasuhisa Matsumoto extracted polysaccharides from *Pholiota nameko* using a hot-water extraction method and found that they exhibited an 86.5% inhibition rate against mouse sarcoma 180. In 2004, Cui Yingjun and colleagues observed through a senescence model in mice that PNP could enhance immune function and possess antioxidant capacity, thereby suggesting their anti-aging properties.^5^ Subsequently, various biological activities of PNP, such as anti-inflammatory, anti-tumor, and lipid-lowering effects, have been continuously discovered. Based on the diverse biological activities of PNP, Li Haiping and colleagues incorporated purified PNP as an additive into yogurt to develop a functional fermented yogurt, reporting the application of these polysaccharides in the field of food additives.^6^ However, focused research on its principal bioactive component—*Pholiota nameko* polysaccharide (PNP)—has accelerated markedly only in the last two decades. Bibliometric analysis (*e.g.*, based on PubMed) shows a notable increase in the annual number of publications related to PNPs since the early 2000s, reflecting their growing recognition as a promising biopolymer in food science, pharmacology, and biomaterials. Commercially, *Pholiota nameko* is a widely cultivated edible mushroom, particularly in East Asia (As shown in the distribution diagram presented in panel C of Fig. 1).^5^ Valued for its delightful taste and rich nutritional content, *Pholiota nameko* possesses a substantial consumer market both domestically and internationally and has become one of the top five commercially cultivated edible mushrooms worldwide.^7,8^ The commercial cultivation of Nameko began in Japan in the 1950s and evolved into automated industrial production by the 1990s. Today, Japan has established a mature industrialized cultivation system for this mushroom. In recent years, China's annual output of Nameko has ranged between 550 000 and 660 000 metric tons, reaching 657 500 metric tons in 2021. In 2020, this industry contributed 1.61% to the total economic value of the sector. The substantial annual output and high degree of industrialization of *Pholiota* microspore highlight both its economic significance and its availability as a raw material for high-value-added products.^9,10^

Despite growing research interest, several key gaps hinder the systematic development and application of PNPs. First, the available literature typically reports fragmented structural information. PNPs do not represent a single entity but rather a family of heteropolysaccharides with diverse molecular weights, monosaccharide compositions, and linkage patterns, characteristics that vary depending on the extraction and purification methods employed.^11,12^ A central challenge lies in correlating this structural complexity with specific functionalities. Second, although numerous studies have reported the bioactivities of crude extracts or specific fractions, there is a noticeable lack of comprehensive and critical discourse that systematically links the fundamental chemical/physical properties of PNPs to their observed biological effects and potential applications. Previous reviews have addressed general types of mushroom polysaccharides or specific aspects of *Pholiota nameko*;^13,14^ however, no review has yet been dedicated to PNPs from an integrated structure-property-function-application perspective.

Therefore, this review aims to specifically address these gaps by compiling existing data and establishing a coherent narrative grounded in the chemical essence of PNPs. We will first systematically integrate current knowledge regarding the chemical and physicochemical fundamentals of PNPs, explaining what PNPs are at the molecular and material levels. This foundation leads into a discussion of extraction and purification strategies. Subsequently, we evaluate the reported bioactivities and their mechanisms, followed by a dedicated and in-depth analysis of structure-activity relationships, which is crucial for rational design. Finally, we assess the applications enabled by these unique properties, concluding with forward-looking perspectives on translating fundamental knowledge into practical innovation.

## 2. Extraction of PNP

The pileus of *Pholiota nameko* presents a yellowish-brown surface with a reddish-brown center, characterized by a smooth, sticky texture and an absence of scales. It typically measures 2.5–8.5 cm in diameter and exhibits a morphology ranging from flattened hemispheric to plane or slightly depressed at the center. Polysaccharides from PNPS are predominantly extracted from the fruiting bodies, although mycelium also serves as a source for certain polysaccharide fractions.^15^ PNP is a heteropolysaccharide, with its monosaccharide composition primarily consisting of glucose, mannose, galactose, and xylose. The respective percentage ranges for each monosaccharide are approximately: glucose (5.3–94.2%), mannose (4.6–59.7%), galactose (5.4–37.2%), and xylose (1.2–5.3%).^16^ It is worth noting that, proteins (0.4–5.4%), and polyphenols (13–16%) are often regarded as impurities during the extraction process and need to be removed in subsequent purification steps to ensure the purity and bioactivity of the target polysaccharides.^16,17^ In Chapter 3, we provide detailed explanations of the methods for removing proteins and reducing pigment. Furthermore, the specific chemical composition varies significantly depending on the extraction method, which directly affects the relative content of each component and the final product's bioactivity. These broad ranges precisely highlight the decisive influence of the extraction process on the chemical characteristics of the final product.

**Fig. 2:**
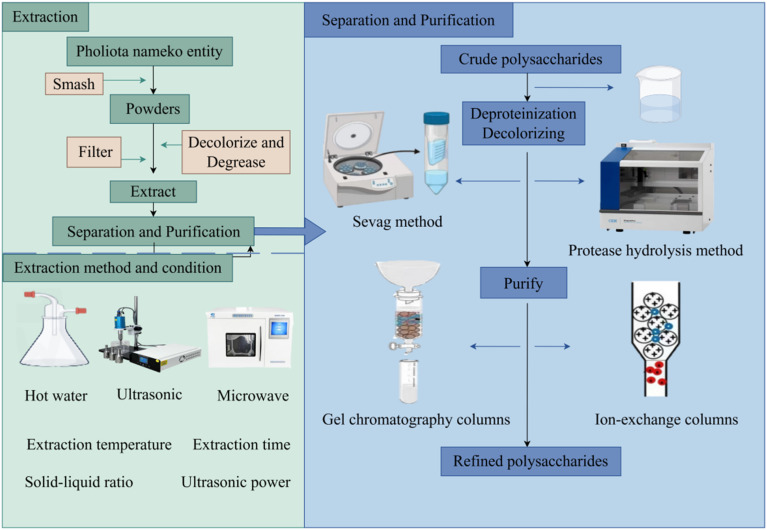
The flow chart of extraction, separation and purification of PNP.

The extraction of polysaccharides from *Pholiota nameko* involves the release of water-soluble components from the insoluble fungal matrix. The general procedure typically includes: (1) raw material pretreatment: dried mushroom fruiting bodies are ground into powder to increase the surface area; (2) solid–liquid extraction: the powdered material is mixed with a solvent (primarily water) and treated under specific conditions to promote the dissolution and diffusion of polysaccharides; (3) solid–liquid separation: insoluble residues are removed by centrifugation or filtration; and (4) concentration and precipitation: the resulting supernatant is concentrated, and polysaccharides are recovered *via* ethanol precipitation, yielding a crude PNP extract. This fundamental framework is common to all extraction techniques, which diverge primarily in the form of energy input and the mechanisms employed to enhance the efficiency of the core extraction step, as illustrated in the left panel of Fig. 2.

The extraction of bioactive polysaccharides from fungi such as *Pholiota nameko* presents several common challenges. A primary difficulty lies in the tough and complex cell wall, a composite structure composed of chitin, β-glucans, and glycoproteins, which constitutes a formidable physical barrier that impedes solvent penetration and the release of intracellular components. Furthermore, the mucilaginous layer covering the mushroom cap adds an external diffusion resistance. A critical yet often overlooked challenge is the structural fragility of the target polysaccharides themselves. The bioactivity of many polysaccharides depends on preserving their native structural features, such as high molecular weight, specific glycosidic linkages (*e.g.*, a β-(1 → 3) backbone), and higher-order conformations (*e.g.*, triple helices). However, the harsh treatments required to disrupt the cell wall—including high temperature, intense shear forces, and extreme pH—can concurrently degrade these delicate structures, leading to loss of bioactivity. This represents a fundamental trade-off between extraction yield and product integrity. Finally, the non-selective nature of the extraction process co-dissolves a complex matrix of proteins, pigments, and small molecules, which complicates downstream purification and may interfere with the accurate assessment of the biological effects of PNP.

Current extraction technologies represent diverse strategies to address these challenges, each exhibiting unique mechanisms, advantages, and limitations in influencing the structural and functional properties of PNP. As summarized in Table 1, which details the extraction conditions, yields, and other relevant factors for each technique, and Table 2, which outlines their respective advantages and limitations.

**Table 1:** Methods and results of extraction of PNP

Extraction method	Optimization factor	Optimization method	Result		References
			Degree of influence	Extraction rate (%)	
Hot water extraction	Extraction temperature: 84 °C	Single-factor experiment	—	(34.0208 ± 0.0433)%	18
	Extraction time: 1.5 h				
	Solid-liquid ratio: 40 : 1				
	Extraction temperature: 90 °C	Response surface experiment	Solid-liquid ratio > extraction time > extraction temperature	(5.24 ± 0.156)%	19
	Extraction time: 3 h				
	Solid-liquid ratio: 25 : 1				
	Extraction temperature: 85 °C	Single-factor experiment, orthogonal experiment	Extraction temperature > solid-liquid ratio > extraction time	24.55 + 0.10%	13
	Extraction time: 2 h				
	Solid-liquid ratio: 40				
	Extraction temperature: 80 °C	Orthogonal experiment	Extraction temperature > extraction time > solid-liquid ratio > ethanol addition multiple	2.69%	15
	Extraction time: 2 h				
	Solid-liquid ratio: 1 : 25				
	Double the amount of ethanol added				
	Extraction temperature: 74.76 °C	Response surface experiment	Extraction temperature > solid-liquid ratio > extraction time	9.05%	20
	Extraction time: 4.80 h				
	Solid-liquid ratio: 1 : 46.65				
	Extraction temperature: 90 °C–100 °C	Single-factor experiment	—	—	21
	Extraction time: 1.5 h				
	Solid-liquid ratio: 1 : 40				
	Extraction times: 1				
	Extraction temperature: 80 °C	Single-factor experiment, orthogonal experiment	—	1.685%	22
	Extraction time: 5 h				
	Solid-liquid ratio: 1 : 60				
	Extraction times: 2.55	Plackett–Burman experiment, response surface methodology	—	24.53%	23
	Ethanol concentration: 95%				
	Ethanol multiple: 3.22				
Ultrasonic-assisted extraction	Ultrasonic time: 40 min	Single-factor experiment, orthogonal experiment	Extraction time > ultrasonic power > solid-liquid ratio > ultrasonic time	13.5%	24
	Ultrasonic power: 700 w				
	Solid–liquid ratio: 22				
	Extraction time: 0.5 h				
	Ultrasonic time: 20 min	Single-factor experiment, orthogonal experiment	Extraction times > ultrasonic power > solid–liquid ratio > ultrasonic time	10.15%	15
	Ultrasonic power: 600 w				
	Solid–liquid ratio: 1/15				
	Extraction times: 0.5 h				
	Ultrasonic time: 16 min	Orthogonal experiment	Ultrasonic time > extraction temperature > ultrasonic power > solid–liquid ratio	9.17%	12,25
	Ultrasonic power: 700 W				
	Solid–liquid ratio: 1 : 30				
	Extraction temperature: 75 °C				
	Ultrasonic time: 15 min	Single-factor experiment, orthogonal experiment	Ultrasonic frequency > ultrasonic time > liquid-to-solid ratio	11.219%	1
	Ultrasonic frequency: 80 Hz				
	Liquid-to-solid ratio: 1 : 20				
Microwave-assisted extraction	Microwave treatment: 4 min	Single-factor experiment, orthogonal experiment	Microwave power > liquid-to-solid ratio > microwave irradiation time > extraction time	14.611%	26
	Liquid-to-solid ratio: 28 : 1				
	Microwave power: 480 w				
	Extraction time: 55 min				
Enzyme-assisted extraction	Solid-liquid ratio: 1 : 5 pH: 4.0	—	—	3.25%	26
	Enzyme dosage (cellulase): 0.7%				
	Enzymolysis time: 120 min				
	Enzymolysis temperature: 55 °C				
	Solid-liquid ratio: 1 : 4 pH: 7.5	—	—	2.45%	26
	Enzyme dosage (neutral protease): 0.4%				
	Enzymolysis time: 130 min				
	Enzymolysis temperature: 45 °C				
	Enzyme dosage: dried *Pholiota nameko* ×0.001	Orthogonal experiment	Extraction temperature > extraction time > water addition multiple > enzymolysis time	2.73%	27
	Enzymolysis time: 45 min				
	Extraction time: 1 h				
	Add water: 15 times				
	Solid-liquid ratio: 1 : 20	Orthogonal experiment	Extraction temperature > extraction time > solid-liquid ratio > extraction time	4.77%	15
	Extraction temperature: 70 °C				
	Extraction time: 1 h				
	Enzymolysis time: 45 min				
	Cellulase (enzyme dosage: 0.05%, pH = 5.0, 45 °C, enzymolysis time: 60 min) trypsin (enzyme dosage: 0.05%, pH = 8.0, 25 °C, enzymolysis time: 45 min) extraction time: 90 min	—	—	Crude polysaccharide (1.404%), pure polysaccharide (0.770%)	28

**Table 2:** Advantages and disadvantages of extraction methods

Extraction method	Advantage	Disadvantage	References
Hot water extraction	It features simple operation, low production cost, high safety, no pollution, mild reaction conditions, minimal use of organic solvents, little damage to the activity and structure of polysaccharides, and is suitable for industrial production	It leads to reduced polysaccharide purity, decreased biological activity, relatively high energy consumption, elevated extraction temperature, low extraction rate, being time-consuming and labor-intensive, as well as low product activity	16, 29, 30 and 31
Alkaline solution extraction method	It results in an increase in free polysaccharides, enhances the polysaccharide extraction rate, ensures that polysaccharides retain a certain level of biological activity, and shortens the extraction time	Destroy the structure of polysaccharides	32, 30 and^31^
Ultrasonic-assisted extraction	It is characterized by low temperature, short processing time of the extraction process, simple operation, solvent saving, fast extraction with high efficiency, no damage to target components, and environmental friendliness	It has certain requirements for equipment and containers, and the extraction time should not be too long, as excessively long extraction time will lead to the destruction of the polysaccharide structure	19, 33 and 32
Microwave-assisted extraction	It boasts short extraction time, high extraction efficiency, low solvent consumption, safety and no pollution, simple operation, good selectivity, and no pollution	It has high energy consumption, tends to destroy the structure of polysaccharides, and easily causes difficulties in the separation of extracts	16 and 30
Enzyme-assisted extraction	It features mild reaction conditions, high polysaccharide activity, shortened extraction time, high yield, easy impurity removal, controllable extraction conditions, and minimal impact on the structure of polysaccharides	It requires harsh conditions, is prone to inactivation, has high demands on experimental equipment, is not suitable for large-scale industrial production, and the enzyme cost is relatively high	16 and 30
High-temperature and high-pressure method	It is simple to operate, requires no addition of any chemical reagents, and is fast and efficient	The control of high temperature is relatively harsh, and it may cause polysaccharide degradation, thereby reducing their biological activity	34–36
Combined use of multiple methods	It features high specificity and efficiency, mild extraction conditions, short polysaccharide extraction time, minimal damage to the polysaccharide structure, and the ability to improve the polysaccharide extraction rate	It may alter the physicochemical properties of polysaccharides and affect their triple-helical conformation	37 and 38

### 2.1 Hot water extraction

Hot water extraction (HWE) is the most commonly used method for isolating polysaccharides from plant and fungal materials. Its principle relies on elevated temperatures to enhance the solubility and diffusion rate of polysaccharides. The advantages of this method include operational simplicity, low cost, the absence of organic solvents, and high safety. However, prolonged exposure to high temperatures may lead to the cleavage of glycosidic bonds, causing polysaccharide degradation, which can reduce molecular weight and alter bioactive conformations. Due to their high polarity, polysaccharides are insoluble in ethanol, facilitating purification through ethanol precipitation.^39^ Extensive research has optimized HWE conditions using single-factor experiments, orthogonal designs, or response surface methodology (RSM). For instance, Chen *et al.* achieved a yield of (34.02 ± 0.04)% under conditions of 84 °C, a liquid-to-solid ratio of 40 : 1 (mL g^−1^), and an extraction time of 1.5 h.^11^ In contrast, You *et al.*, through RSM optimization, reported a yield of (5.24 ± 0.156)% when extracting at 90 °C with a liquid-to-solid ratio of 25 : 1 for 3 h.^19^ Similarly, Zhu Zhenyuan *et al.* employed single-factor and orthogonal experimental designs to determine the optimal HWE parameters for PNP: a temperature of 85 °C, an extraction time of 2 h, and a solid-to-liquid ratio of 1 : 40 (g mL^−1^), ultimately obtaining a polysaccharide yield of 24.55 ± 0.10%.^13^ The polysaccharide yields reported across different studies show significant variation, ranging from 5.24% to 34.02%. These discrepancies may be attributed to differences in raw material pretreatment, polysaccharide quantification methods, or experimental design, highlighting the necessity for standardized protocols in polysaccharide extraction research. Although HWE can provide relatively high yields, researchers must weigh the potential negative impact of high temperature on the structural integrity of PNP, particularly when the target bioactivity depends on high molecular weight or specific conformations such as the triple-helical structure.

### 2.2 Alkaline solution extraction method

Alkaline extraction utilizes dilute alkaline solutions to disrupt the physicochemical interactions within plant cell walls^40^ and alters the binding interactions between polysaccharides and proteins, thereby facilitating polysaccharide release. While effective, alkaline conditions may induce chemical modifications—such as deacetylation or elimination—that irreversibly alter the structure and properties of polysaccharides. In a study by Zhao Xiaolin, alkaline extraction with a 0.5 mol L^−1^ sodium hydroxide solution, followed by freeze-drying, yielded 19.4 g of alkaline-extracted *Pholiota nameko* polysaccharide (APNP), corresponding to an extraction efficiency of 0.5%.^41^ This result is significantly lower than those obtained with other extraction methods, which may be attributed to the potential degradation of polysaccharide structures under alkaline conditions.

### 2.3 Ultrasonic-assisted extraction

Ultrasonic-assisted extraction (UAE) has emerged as an efficient and practical technique for extracting polysaccharides. This method leverages the mechanical effects and acoustic cavitation generated by ultrasound to disrupt biological structures such as cell walls and membranes, while significantly enhancing intracellular mass transfer and accelerating the release of polysaccharides.^42^ Compared with HWE, UAE typically achieves higher yields within shorter timeframes and at lower temperatures.^43^ For instance, Wang *et al.* optimized ultrasonic treatment time, power, liquid-to-solid ratio, and subsequent water-bath duration using a combination of single-factor and orthogonal experimental designs. Under the optimized conditions of 700 W ultrasonic power, 40 min of sonication, and a liquid-to-solid ratio of 22 : 1, followed by a 0.5 h water-bath extraction, a yield of 13.5% was obtained.^24^ However, studies have indicated that while ultrasound can markedly increase the extraction rate of PNP, it may also lead to reduced polysaccharide purity. This phenomenon could be attributed to the co-extraction of non-polysaccharide components or the degradation of high-molecular-weight polysaccharides into lower-molecular-weight saccharides under intense ultrasonic treatment.^26^ Furthermore, UAE carries a potential risk of damaging polysaccharide structures,^40^ necessitating strict control of extraction parameters to avoid degradation. In summary, UAE represents an efficient extraction method but should be carefully optimized to balance yield and structural integrity.

### 2.4 Microwave – assisted extraction

Microwave-assisted extraction (MAE) utilizes microwave irradiation to induce rapid dielectric heating of polar molecules within cells, uniformly raising intracellular temperature and pressure, which leads to cell rupture and promotes the release of intracellular polysaccharides. This technique offers notable advantages such as short processing time and reduced solvent consumption.^44^ Li Dehai *et al.* optimized the MAE process through a combination of single-factor experiments and orthogonal array design. The optimized conditions—microwave power of 480 W, irradiation time of 4 min, liquid-to-solid ratio of 28 : 1, followed by a 55-min water-bath extraction—yielded a polysaccharide recovery rate of 14.61%.^45^ Among the investigated factors, the order of influence on extraction yield was: microwave power > liquid-to-solid ratio > microwave irradiation time > water-bath extraction time.

Microwave power proved to be the most influential factor affecting the yield. Similar to UAE, the high-energy environment of MAE may also lead to polysaccharide degradation. However, due to its extremely short treatment duration, MAE could potentially cause less structural damage compared to prolonged HWE. MAE is suitable for rapid extraction but requires specialized equipment, and achieving uniform heating must be addressed when scaling up the process.

### 2.5 Enzyme-assisted extraction

Enzyme-assisted extraction (EAE) facilitates the release and extraction of polysaccharides from biological samples under mild conditions by utilizing specific enzymatic reactions, such as those catalyzed by cellulases and proteases. With advantages including high efficiency in impurity removal and favorable recovery rates, EAE represents a promising extraction technique.^46^ In studies on EAE, Zhao *et al.* systematically screened enzymes and determined that hydrolysis with cellulase (solid-to-liquid ratio 1 : 5, pH 4.0, enzyme dosage 0.7%, 55 °C for 120 min) yielded 3.25% PNP, which was higher than the 2.45% obtained using neutral protease (solid-to-liquid ratio 1 : 4, pH 7.5, enzyme dosage 0.4%, 45 °C for 120 min), indicating the superior efficacy of cellulase.^26^ Liu *et al.* optimized a process employing 0.1% enzyme (cellulase/trypsin), a water-to-solid ratio of 15 : 1, enzymatic hydrolysis for 45 min, followed by hot-water extraction at 90 °C for 1 h, achieving a yield of 2.73%.^27^ Similarly, Shi Jing *et al.* increased the yield to 4.77% by applying cellulase hydrolysis for 45 min (solid-to-liquid ratio 1 : 20, extraction at 70 °C for 1 h).^15^ The yields reported for EAE (ranging from 2.45% to 4.77%) are generally lower than those achieved by physical methods, and the cost of enzymes remains relatively high. However, the high specificity of enzymatic reactions allows intracellular polysaccharides to be released in the mildest manner, thereby better preserving the structural integrity of the extracted polysaccharides. This approach is particularly suitable for extracting bioactive polysaccharides that are sensitive to heat and shear stress. Furthermore, the selection of enzymes is critical and should be based on the composition of the cell wall and the form in which the target polysaccharides exist (*e.g.*, whether they are bound to proteins).

### 2.6 High – temperature and high – pressure method

High-temperature and high-pressure extraction enhances the aqueous solubility of target compounds and accelerates solvent diffusion by elevating both temperature and pressure, offering advantages such as reduced solvent consumption and shortened extraction time.^47^ In a representative high-temperature and high-pressure extraction process, 100 g of dried *Pholiota nameko* was soaked in 900 mL of distilled water at 25 °C for 12 h, followed by extraction at 110 °C and 1.2 MPa for 30 min, resulting in a crude polysaccharide yield of 41.2%.^47^ This yield is notably higher than those achieved by most conventional methods, and the extraction time is significantly reduced. However, the method demands equipment with high pressure and temperature resistance, which to some extent limits its large-scale application.

### 2.7 Combined use of multiple methods

To overcome the limitations of individual methods, combined strategies have been developed accordingly. Song Yuguang *et al.* achieved a significant increase in polysaccharide yield by employing a sequential process involving enzymatic hydrolysis followed by hot water extraction. Specifically, the protocol consisted of initial enzymatic treatment with cellulase (0.05% w/w, pH 5.0, 45 °C) for 60 min, followed by further hydrolysis with trypsin (0.05% w/w, pH 8.0, 25 °C) for 45 min, and concluding with hot water extraction at 80 °C for 90 min. This combined approach increased the yield of PNP by 38% and 67%, respectively, compared to enzymatic treatment or hot water extraction alone.^28^ Other emerging hybrid extraction technologies include integrated ultrasonic-cold plasma extraction, pressurized liquid extraction coupled with enzyme-assisted extraction,^48^ and natural deep eutectic solvents (NADES) combined with ultrasound-assisted extraction,^49^ among others. For instance, in the extraction of bioactive compounds from licorice roots, the combined application of ultrasound and cold plasma technology significantly enhanced the concentrations of total phenols, flavonoids, and glycyrrhizic acid, along with elevated antioxidant activity, compared to traditional maceration or single-method extraction. Specifically, increases of 13.29% in total phenols, 42.41% in flavonoids, and 15.89% in glycyrrhizic acid were observed relative to conventional maceration. This demonstrates the potential of hybrid techniques as innovative and efficient extraction platforms.^50^ These integrated approaches achieve higher efficiency and superior structural preservation by sequentially targeting different extraction barriers, representing a promising direction for the future development of extraction technologies.

### 2.8 Analysis of extraction methods

Different techniques for extracting PNP essentially represent distinct trade-offs between “efficiency” and “structural preservation.” If the goal is to obtain immunomodulatory polysaccharides with specific conformations (*e.g.* triple helices), priority should be given to mild methods such as EAE or optimized UAE/MAE. Conversely, if high yield is desired for applications such as food thickeners, HWE or high-temperature/high-pressure extraction may be more suitable. Additionally, environmental friendliness and cost considerations must also be weighed when scaling up for industrial production.

The future direction of technological development lies in the synergistic integration of different mechanisms. This can be achieved, for example, through a sequential strategy involving gentle enzymatic or physical disruption of cell walls followed by brief, low-intensity physical field-assisted extraction. Such an approach aims to ensure satisfactory yield while precisely preserving the key bioactive structures of PNP.

## 3. Isolation and purification of PNP

Following extraction, the obtained crude PNP is a complex mixture comprising target polysaccharides along with various impurities such as proteins, pigments, low-molecular-weight sugars, and inorganic salts. The physicochemical properties of this system are primarily characterized by high hydrophilicity,^51^ a broad molecular weight distribution (polydersonality), and significant structural heterogeneity. Furthermore, there may be an overlap in properties such as solubility and charge with certain impurities (*e.g.* glycoproteins or polyphenols).^52^ These characteristics dictate that the core of crude polysaccharide purification lies in achieving selective separation based on differences in molecular size, charge, solubility, or specific chemical interactions between the target polysaccharides and various impurities, while minimizing the loss of polysaccharides and preserving their native bioactive conformation.^53^ The principle of deproteinization is based on the differences in solubility in organic solvents or reactivity with certain chemical reagents between proteins and polysaccharides. Accordingly, common purification methods include the Sevag method, TCA (trichloroacetic acid) method, and salting-out method.^54^ The removal of pigments typically relies on the physical adsorption or chemical binding of pigment molecules to specific adsorbents. Standard techniques for this step include activated charcoal adsorption and macroporous resin adsorption.^55,56^ Low-molecular-weight impurities (such as low-molecular-weight sugars and inorganic salts) can be removed by methods including dialysis and ultrafiltration.^57^ Therefore, effective purification is a critical step for obtaining polysaccharide fractions suitable for accurate structural characterization, reliable bioactivity assessment, and high-value applications. The structural information of PNP resulting from different extraction and purification methods is summarized in Table 3. The main challenges in purifying PNP stem from its inherent polydispersity (broad molecular weight range), structural heterogeneity (diverse monosaccharide compositions and branching patterns), and potential similarity in physicochemical properties to certain contaminants (*e.g.* other glycoproteins or polyphenols). Purification strategies must achieve separation based on molecular size, charge, solubility, or specific chemical interactions, while minimizing polysaccharide loss and preserving their native bioactive conformation.

**Table 3:** Extraction and structure information of PNP

Name	Extraction method	Purification method	Yield	Purity	Molecular weight	Methods	Structural features	Molar ratio of monosaccharide composition	References
APNP-A-b	Alkaline solution extraction method (0.5 mol L^−1^ NaOH solution; solid–liquid ratio: 1 : 20 (w/v); pH: 7.0; concentrated under reduced pressure at 60 °C)	Sephadex G-100 chromatography column	43.4%	—	1.65 × 10^4^ Da	IR, NMR, IC, methylation analysis, periodate oxidation, enzymatic hydrolysis	Main chain: β-1,6-d-Glcp; side chains: 1,3-d-Glcp, *t*-d-Glcp, and a small amount of GlcA linked to 1,3-d-Glcp, which is a β-1,6-glucan structure	Glc : Gal : Man : GlcA : Xyl = 79.4 : 6.5 : 5.9 : 7.0 : 1.2	41
APNP-N-b		Sepharose CL-6B gel filtration chromatography column	72.8%	—	2.14 × 10^4^ Da	IR, NMR, IC, methylation analysis, periodate oxidation, enzymatic hydrolysis	Main chain: α-1,6-d-Galp; side chains: *t*-β-d-Manp and a very small amount of β-1,3-d-Manp	Glc : Gal : Man : Me-Gal : Xyl : Fuc = 17.9 : 34.9 : 30.5 : 12.4 : 2.0 : 2.3	41
APNP-A-c		Sephadex G-100 chromatography column	31.0%	—	4.2 × 10^3^ Da	IR, NMR, IC, methylation analysis, periodate oxidation, enzymatic hydrolysis	Main chain: β-1,6-d-Glcp; side chains: 1,3-d-Glcp and *t*-d-Glcp, with a small amount of GlcA linked to the 1,3-d-Glcp side chains, and it is a β-1,6-glucan structure	Glc : Gal : Man : GlcA : Xyl = 90.6 : 1.9 : 1.3 : 4.5 : 1.6	41
WBSP	Alkaline solution extraction method (3 L 0.125 mol L^−1^ NaOH, 0.05% NaBH_4_ (3L, 25 °C, 2 h))	DEAE-cellulose ion exchange column chromatography	—	84.2%	3.578 × 10^5^ Da	XRD, NMR, HPSEC-MALLS, ultraviolet-visible spectroscopy	Main chain: → 3)-β-d-Glcp-(1 →; side chain: → 6)-β-d-Glcp-(1 →	Xyl : Man : Glc : Gal = 3.1 : 15.6 : 75.9 : 5.4	16
SASP	Alkaline solution extraction method (1.25 mol L^−1^ NaOH, 0.05% NaBH_4_ (3 L, 2 h))	DEAE-cellulose ion exchange column chromatography	—	95.2%	8.45 × 10^6^ Da	XRD, NMR, HPSEC-MALLS, ultraviolet-visible spectroscopy	Main chain: → 3)-β-d-Glcp-(1 →; side chain: → 6)-β-d-Glcp-(1 →	Xyl : Man : Glc = 1.2 : 4.6 : 94.2	16
BWSP-1	Hot water extraction (Distilled water, solid–liquid ratio: 1 : 30 (w/v), extraction at 100 °C for 2 hours, centrifugation (8000 rpm, 10 min)	DEAE-cellulose ion exchange column chromatography	—	96.53%	6.99 × 10^5^ Da	IR, NMR, XRD, GC-MS and methylation analysis	The main chain is composed of → 6)-β-d-Manp-(1 →, → 3,6)-β-d-Manp-(1 →, and → 4)-α-d-Glcp-(1 →; a branched chain β-d-Xylp-(1 → is linked to the O-3 position of → 6)-β-d-Manp-(1 →	Xyl : Man : Gal = 0.235 : 1 : 0.117	16
BWSP-2		DEAE-cellulose ion exchange column chromatography	—	94.06%	7.78 × 10^5^ Da	IR, NMR, XRD, GC-MS and methylation analysis	Main chain: Composed of → 3,6)-β-d-Manp-(1 →, → 6)-d-Manp-(1 →, and → 4)-β-d-Manp-(1 →; side chain: → 4)-β-d-Xylp-(1 → and the O-3 position of → 3,6)-β-d-Manp-(1 →	Xyl : Man : Glc = 0.262 : 1 : 0.096	16
wPNP-a1	Hot water extraction((1 : 16 (w/v), 90 °C, 2.5 h, ×3))	Sephadex G-150 gel chromatography	—	65. 17%	419 310 Da	HPLC, FTIPR, GC-MS, NMR, SEM, AFM	The glycosidic bonds are of β-type, no acetyl groups are present, and it exhibits the characteristic infrared absorption peaks of common polysaccharides	Xyl : Man : Gal : Glc = 3.91 : 2.77 : 1.91 : 1	29
PNP	Hot water Extraction(H_2_O, 1 : 40 (w/v), 80 °C, 2 h, ×3)	Sephadex G-200	24.55%	95.29%	1.89 × 10^3^ kDa	Ultraviolet-visible spectroscopy, FT-IR, NMR, GC-MS, methylation analysis	Main chain: → 3)-Glc-(1 →, → 3,6)-Glc-(1 →, → 3)-Man-(1 →, and → 3,6)-Man-(1 →	Glc : Man = 4.24 : 1.00	58
WPNP-A-a	Hot water extraction (double extraction (H_2_O 1 : 10 (w/v), 100 °C 4 h; H_2_O 1 : 8 (w/v), 100 °C 3 h))	DEAE-cellulose column chromatography, gel filtration chromatography	57.6%		1.1 × 10^6^ Da	NMR, methylation anal	Main chain: 1,3-Man; at the O-3 position, it is linked to 1,3-Glc or terminal *t*-Xyl, and some man molecules are bound to *t*-Xyl at the O-2 and O-6 positions	Man : Xyl : GlcA : Glc = 61.9 : 20.4 : 14.2 : 3.5	59
WPNP-N-b	Water extraction and ethanol precipitation method (double extraction (H_2_O 1 : 10 (w/v), 100 °C 4 h; H_2_O 1 : 8 (w/v), 100 °C 3 h))	DEAE-cellulose column chromatography, gel filtration chromatography	43.3%		1.4 × 10^4^ Da	NMR, methylation anal	Main chain: α-1,6-d-Galp, with a small amount of α-1,6-O-Me-d-Galp structure, and substitution occurs at the O-2 position of some gal units; side chain: composed of *t*-β-d-Manp	Gal : Man : Glc : Me-Gal : Xyl = 65.1 : 24.2 : 4.9 : 4.6 : 1.2	59
GHW-PN	Hot water extraction (100 °C, 6 h), H_2_O 1L at 25 °C, 6 h (×3) C_2_H_6_O (3 : 1 v/v) centrifugation, dialysis, H_2_O 1L at 100 °C, 6 h (×3) C_2_H_6_0 (3 : 1 v/v), centrifugation, dialysis	A-amylase treatment (86 °C, 4 h)	1.46%	—	—	NMR, methylation anal	The main chain is composed of (1 → 3)-linked β-d-Glcp units, and its O-6 positions are highly substituted by either single β-d-Glcp units or (1 → 6)-linked β-d-Glcp side chains	Glc = 100%	60
PNP	Ultrasonic-assisted extraction (Ultrasound power: 97 W, ultrasound duration: 13.5 min, and liquid-to-material ratio: 20.5 : 1 (80 °C, 3 h))	DEAE Sepharose fast flow ion-exchange cellulose chromatography column	3.42%	93.76%	20199 Da	GC, IR, GC-MS, NMR, partial acid hydrolysis, smith degradation, periodate oxidation	It contains three types of glycosidic bonds: 1→, 1 → 3, and 1 → 6	d-Man : d-Glc : d = 3.56 : 12.2 : 1	61
WPNP-N-b	Water extraction and ethanol precipitation method (Double extraction (H_2_O 1 : 10(w/v), 100 °C 4 h; H_2_O 1 : 8 (w/v), 100 °C 3 h))	DEAE-cellulose column chromatography, gel filtration chromatography	43.3%		1.4 × 10^4^ Da	NMR, methylation anal	Main chain: α-1,6-d-Galp, with a small amount of α-1,6-O-Me-d-Galp structure, and substitution occurs at the O-2 position of some gal units; side chain: composed of *t*-β-d-Manp	Gal : Man : Glc : Me-Gal : Xyl = 65.1 : 24.2 : 4.9 : 4.6 : 1.2	59
CW	Cold water extraction, CHCIg-MeOH (2 : 1 v/v, 60 °C, 5 h, 3×)	—	16.55%	—	—	GC-MS analysis, NMR, methylation anal	—	Man : Gal : Glc = 14.1 : 6.3 : 79.6	62
SCW	Cold water extraction, H_2_O at 25 °C, 6 h, 3×	EtOH precipitation and freeze and thawing	47.86%	—	—	GC-MS analysis, NMR, methylation anal, rheological measurements, controlled smith degradation of -d-glucan	—	Man : Gal : Glc = 14.1 : 6.3 : 79.6	62
bG-PN	Cold water extraction (H_2_O at 25 °C, 6 h, 3×, EtOH precipitation and freeze and thawing, treatment with fehling solution)	Freeze and thawing process	43.33%	—	—	GC-MS analysis, NMR, methylation anal	The main chain is composed of Glcp-(1 → 3) linkages, and its O-6 positions are highly substituted by Glcp residues and/or Glcp-(1 → 6)-linked side chains	Glc = 100%	62
CPPN	Ultrasonic-assisted extraction (1 : 7, w : v), sonicate (300 W, 30 min), hold (90 °C, 4 h)	Cellulose column and Sephadex G-100	88.3%	—	2.26 × 10^4^ Da	NMR, FT-IR	—	Man : Rha : Glc : Gal : Xyl : Ara = 1.75 : 5.11 : 38.21 : 10.53 : 18.72 : 25.68	63 and 64
MZPS	Water extraction and alcohol precipitation(90 °C, 2 h)	DEAE-52 column chromatography, Sephadex G-100 column chromatography	—	—	36.42 Da	HPGPC, HPLC	—	Glc : Gal : Man : Ara : GlcA : GalA = 110.31 : 6.51 : 1.93 : 1.11 : 1.00	65
MZPS-1	Water extraction and alcohol precipitation (90 °C, 2 h)	DEAE-52 cellulose anion exchange chromatography (0 mol L^−1^ NaCl), Sephadex G-100 gel permeation chromatography	—	—	55.31 kDa	FTIR, UV spectroscopy analysis	—	Glc : Man : Gal : Ara = 101.52 : 1.83 : 1.31 : 1.00	65
MZPS-2	Water extraction and alcohol precipitation (90 °C, 2 h)	DEAE-52 cellulose anion exchange chromatography (0.3 mol L^−1^ NaCl), Sephadex G-100 gel permeation chromatography	—	—	13.63 kDa	FTIR, UV spectroscopy analysis	—	Glc : Man : Gal : GalA : Ara = 172.59 : 5.29 : 4.61 : 4.20 : 1.01 : 1.00	65

A typical purification strategy follows the chemical logic of “impurity removal first, followed by fractionation” (as illustrated in the right panel of Fig. 2). It initially involves deproteinization and decolorization to remove the majority of non-polysaccharide impurities. Subsequently, fractionation of the polysaccharide mixture is conducted using ion-exchange chromatography (separating by charge) or gel-filtration chromatography (separating by molecular size) to obtain structurally homogeneous fractions. This lays a solid foundation for subsequent precise structural characterization and structure-activity relationship studies. The key techniques applied in PNP purification are detailed in the following sections.

### 3.1 Deproteinization and decolorization of PNP

Residual protein impurities present during polysaccharide extraction can interfere with structural characterization and distort the evaluation of biological activities, potentially leading to misinterpretation of structure-activity relationships. Therefore, deproteinization is an essential preparatory step prior to the analysis of PNP.^66^ Current deproteinization techniques can be categorized into chemical, physical, and biological methods. Chemical approaches include the Sevag method,^67^ TCA precipitation,^68^ and the trifluorotrichloroethane method.^69^ Physical methods involve the use of anion-exchange resins,^70^ while biological strategies employ enzymatic treatments such as proteases.^54^ In recent investigations, the Sevag method has been predominantly applied for deproteinizing PNP. Through orthogonal experimental optimization, Chen Xiaoning *et al.* established optimal conditions for extracellular PNP deproteinization: a chloroform-to-*n*-butanol volume ratio of 6 : 1, a Sevag reagent-to-sample solution ratio of 3.5 : 1, and four extraction cycles. Under these conditions, the protein removal rate reached 91.37%, with a polysaccharide loss of 23.58%. For intracellular PNP, the optimal parameters were a chloroform-to-*n*-butanol ratio of 6 : 1, a reagent-to-sample ratio of 2.5 : 1, and five extraction cycles, achieving a protein removal rate of 62.59% and a polysaccharide loss rate of 22.22%. The influence of factors on deproteinization efficiency followed the order: Sevag reagent dosage > chloroform-to-*n*-butanol ratio > number of extraction cycles.^18^

Residual pigments not only compromise the bioactivity of polysaccharides but can also form irreversible adsorptions with cellulose resins, thereby severely impairing subsequent chromatographic purification processes. Common decolorization techniques for polysaccharide preparations include activated carbon adsorption and oxidative treatment.^71^ Macroporous resins are widely used in separation and purification processes.^72^ Li Dehai *et al.* employed such resins to purify PNP and compared the decolorization performance of three resins—ADS-5, AB-8, and D101—with AB-8 exhibiting superior efficiency.^73,74^ The optimal purification conditions using AB-8 resin were determined as follows: adsorption time of 3 h, pH 5.0, sample concentration of 1.5 mg mL^−1^, 70% ethanol as eluent, desorption time of 4 h, and flow rate of 2 mL min^−1^. Under these conditions, an adsorption rate of 86.67% and a desorption rate of 71.38% were achieved.^74^

### 3.2 Purification of PNP

Common techniques for the further purification of PNP include column chromatography^75^ and fractional precipitation.^76^ Among fractional precipitation methods, quaternary ammonium salt precipitation is widely employed. This method facilitates the formation of insoluble complexes with acidic polysaccharides, enabling their separation from neutral polysaccharides. Guo Guoyun *et al.* applied the quaternary ammonium salt precipitation method to crude PNP through a series of steps—dissolution, precipitation, redissolution, dialysis, and freeze-drying—yielding three distinct subfractions: an acidic polysaccharide fraction (wPNP-a), a neutral fraction (wPNP-b), and a basic fraction (wPNP-c).^29^ Column chromatography serves as a fundamental purification approach and includes ion-exchange chromatography and gel filtration chromatography. Anion-exchange chromatography is particularly prevalent for polysaccharide purification and is effective in separating acidic polysaccharides, neutral polysaccharides, and mucopolysaccharides. Commonly used anion exchangers include diethylaminoethyl (DEAE)-cellulose, DEAE-Sephadex, and DEAE-Sepharose. Using a DEAE-Sepharose Fast Flow column, crude PNP was separated into two polysaccharide fractions: a neutral polysaccharide, designated PNP-1 (yield 8.68%), and an acidic polysaccharide, PNP-2 (yield 41.20%). Both fractions were confirmed to be homogeneous through Sephadex G-100 gel filtration and high-performance gel permeation chromatography (HPGPC).^29^ Similarly, Xiong Shanqiang *et al.* purified hot water-extracted crude polysaccharide (BWSP) using DEAE-cellulose anion-exchange chromatography, obtaining two purified fractions, BWSP-1 and BWSP-2. HPGPC analysis revealed that both components exhibited relatively single and symmetric narrow peaks, indicating high purity.^16^ Gel filtration chromatography separates polysaccharides based on differences in molecular size and shape. Commonly used media include Sephadex (*e.g.*, Sephadex G series, Sephadex LH-20), Sepharose (agarose-based gel), Cellufine GCL-2000 (cellulose gel), and Bio-Gel P (polyacrylamide gel). Guo Guoyun *et al.* further purified the predominant acidic subfraction wPNP-a—obtained previously *via* quaternary ammonium salt precipitation—using a Sephadex G-150 column, yielding a homogeneous fraction designated wPNP-a1. This fraction displayed a single symmetric peak in HPGPC, with a molecular weight of 419 310 Da.^29^ In another study, Zhao Xiaolin *et al.* purified alkali-extracted neutral PNP (APNP-N) using a Sepharose CL-6B preparative column, obtaining APNP-N-b with a yield of 72.8%. For the alkali-extracted acidic PNP (APNP-A), purification *via* Sephadex G-100 gel column yielded two subfractions, APNP-A-b and APNP-A-c, with yields of 43.4% and 31.0%, respectively.^41^

For the deproteinization, decolorization, and purification of PNP, appropriate techniques must be selected according to the target product's required attributes, such as purity, molecular weight, and charge properties. Chemical methods, though efficient, may compromise the bioactivity of PNP, whereas physical and biological approaches are generally milder but often involve higher operational costs. Commonly employed deproteinization methods for polysaccharides include the TCA method, trifluorotrichloroethane treatment, anion-exchange resin adsorption, protease digestion, and ultrasound-assisted deproteinization. Among these, the TCA method may be particularly suitable for PNP purification. For instance, Xie *et al.* compared the Sevag and TCA methods for deproteinizing crude polysaccharides from *Fomitopsis baumii* and found that the TCA method achieved a higher protein removal rate (82%) under optimized conditions, with a polysaccharide loss of only 10.8%.^33^ Additional decolorization strategies include hydrogen peroxide treatment. Yu *et al.* optimized the decolorization of alkali-soluble polysaccharides from *Chrysanthemum indicum* using hydrogen peroxide, which provided effective pigment removal with minimal polysaccharide loss, thereby preserving polysaccharide integrity.^77^ However, specific decolorization conditions for PNP require further experimental optimization. Beyond conventional techniques such as anion-exchange chromatography, gel filtration, and quaternary ammonium salt precipitation, molecularly imprinted technology (MIT) has emerged as a promising tool for final-stage purification. This novel approach allows the synthesis of starch-imprinted magnetic nanoparticles (MMIPs), which demonstrate a starch adsorption capacity of 15.45 mg g^−1^ and exhibit superior selectivity for starch over other carbohydrates. MIT is particularly suitable for rapid purification of polysaccharides from traditional Chinese medicines (TCMs),^78^ and its application is anticipated to greatly enhance the purification efficiency of PNP.

## 4. Structural analysis of PNP

### 4.1 Rheological properties

Aqueous solutions of PNP exhibit non-Newtonian fluid behavior, characterized by shear-thinning where the apparent viscosity decreases with increasing shear rate.^60,62,79^ For instance, PNP fractions obtained through sequential cold-water and hot-water extraction (designated as CW-PNPs and HW-PNPs, respectively) demonstrate pseudoplastic behavior under varying mass concentrations, temperatures, metal ion types, and pH conditions. The rheological properties of both CW-PNPs and HW-PNPs conform to the power-law model.^79^ At a concentration of 1% (w/w), the apparent viscosity follows the order: HW-PNPs > CW-PNPs. Furthermore, temperature, pH, and salt ions influence the non-Newtonian characteristics of these fractions to varying degrees.^60,79^

Regarding dynamic rheological properties, frequency sweep tests conducted at varying concentrations (*e.g.* 6, 8, 10, 12 mg mL^−1^ in one study and 2, 4, 10, 15 mg mL^−1^ in another) reveal that both the storage modulus (*G*′) and loss modulus (*G*″) of PNP solutions increase with frequency. Notably, at concentrations of 10 mg mL^−1^ and above, *G*′ exceeds *G*″ across the entire measured frequency range without observable crossover points, indicating that concentrated PNP solutions exhibit weak gel-like behavior.^16,32^ The shape of the frequency sweep curves is significantly influenced by external factors. Elevated temperature leads to a substantial decrease in both moduli, shifting the entire curve downward and weakening the gel structure, making the solution behavior more liquid-like.^32^ The effects of salt ions are complex; the addition of NaCl or CaCl_2_ can cause either an increase or decrease in apparent viscosity at low shear rates, while a consistent decrease is observed at high shear rates.^16^

Comparatively, the rheological profile of PNP offers distinct advantages over some common food thickeners. Traditional agents like xanthan gum, despite high viscosity and pronounced shear-thinning, can degrade at high temperatures (100–120 °C) and are sensitive to ionic strength.^80^ Gelatin, while forming strong thermoreversible gels, has a relatively low melting point (*e.g.* ∼22 °C for fish gelatin) and is animal-derived.^81^ In contrast, certain PNP fractions demonstrate excellent thermal stability and reversible gelation. For instance, a polysaccharide from *Pholiota nameko* (GHW-PN) showed a 19-fold increase in zero-shear viscosity when concentration rose from 0.5% to 2%.^82^ Its gel network remained stable over multiple temperature cycles between 5 °C and 80 °C, indicating resilience to common food thermal processing.^83^ This thermal stability, combined with inherent bioactivities such as anti-inflammatory properties, positions PNP not only as a texture-modifying agent but also as a potential functional food additive that can enhance product health value.

### 4.2 Thermal stability

PNP demonstrates favorable thermal stability across a relatively broad temperature range.^62^ Thermogravimetric analysis (TGA) indicates that the thermal decomposition of PNP occurs predominantly in three stages: below 200 °C, loss of free and bound water occurs; between 200 and 400 °C, substantial mass loss is observed due to polysaccharide decomposition; and above 400 °C, the decomposition stabilizes, with the mass loss rate decreasing significantly.^32^ However, thermal stability varies significantly among PNP fractions obtained through different extraction methods. Specifically, five sequential fractions were isolated from decolorized and defatted *Pholiota nameko* fruiting bodies: room temperature water-soluble polysaccharides (RTSP), hot water-soluble polysaccharides (BWSP), high-pressure extracted polysaccharides (HPSP), and two alkali-extracted fractions using 0.125 mol L^−1^ NaOH (WBSP) and 1.25 mol L^−1^ NaOH (SASP). Quantitative TGA data indicate that the alkali-extracted fractions (WBSP and SASP) possess higher thermal stability. Their main decomposition stage occurs at approximately 270–320 °C, with a mass loss exceeding 60% in this range. Furthermore, the temperature at which the mass loss rate begins to decelerate notably on the derivative thermogravimetry (DTG) curve is lower for these fractions, suggesting a more gradual degradation process.^16^ In contrast, the water-extracted fractions (RTSP, BWSP, HPSP) demonstrate lower thermal stability. This difference is attributed to the higher content of short-chain sugars and highly branched polysaccharides in WBSP and SASP, which adopt loose and amorphous structural arrangements that facilitate thermal degradation.^16^

### 4.3 Molecular weight

Molecular weight serves as a critical parameter for elucidating the chemical structure and physicochemical properties of polysaccharides, and plays an essential role in establishing structure-activity relationships. Common techniques for determining the molecular weight of polysaccharides include chromatographic methods, mass spectrometry, and viscometry.^84,85^ Currently, chromatographic techniques are predominantly employed for the determination and analysis of the molecular weight of PNP. These include high-performance size-exclusion chromatography coupled with multi-angle laser light scattering (HPSEC-MALLS),^16^ high-performance gel permeation chromatography (GPC),^1^ and high-performance liquid chromatography (HPLC).^58^

Due to the complex and heterogeneous microstructure of polysaccharide molecules, techniques such as GPC or HPLC are typically employed for preliminary separation to obtain homogeneous polysaccharide fractions. These fractions generally appear as single, sharp, and symmetrical peaks in chromatograms. The polydispersity index (PDI) is used to evaluate the uniformity of polymer molecules and is numerically equal to the weight-average molecular weigh (Mw) divided by the number-average molecular weight (Mn). In chromatograms, the sharper the peak, the closer the polydispersity index of the polysaccharide is to 1, reflecting its higher homogeneity.^86^ Both GPC and HPLC determine molecular weight by means of pre-established standard curves, offering the advantages of operational simplicity and high analytical efficiency. For instance, Xiang Ying^61^ utilized BreezGPC software to develop a regression model between the logarithm of the molecular weight of dextran standards and their elution volume, and calculated the peak molecular weight (Mp) of PNP to be 20.5 kDa. The Mw was 20 199 Da, the Mn was 19 437 Da, and the PDI was 1.039, indicating high structural homogeneity of PNP. In another study, Han Dan^58^ constructed a standard curve based on the logarithm of molecular weight *versus* retention time for various dextran standards, and determined the molecular weight of alkali-extracted neutral PNP (APNP) to be 1.89 × 10^6^ Da according to its elution time. Xiong Shanqiang^16^ isolated five distinct fractions (RTSP, BWSP, HPSP, WBSP, and SASP) from PNP and analyzed their molecular weights using HPSEC-MALLS. The molecular weights were found to be 3.58 × 10^2^ kDa for WBSP (PDI: 2.037) and 8.45 × 10^3^ kDa for SASP (PDI: 1.593). Furthermore, BWSP was separated into two subfractions (BWSP-1 and BWSP-2), with molecular weights of 6.99 × 10^2^ kDa and 7.78 × 10^2^ kDa, respectively. It can be concluded that different homogeneous fractions of PNP exhibit distinct molecular weights. Moreover, even for the same fraction, the measured molecular weight is highly dependent on the separation and purification protocols employed, as well as the methodologies used for molecular weight determination and calculation.

### 4.4 Monosaccharide composition

Monosaccharide composition constitutes a fundamental aspect of the primary structure of polysaccharides, and its analysis is essential for elucidating the relationship between structural features and biological activities.^77^ Current analytical approaches for determining the monosaccharide composition of fungal polysaccharides typically involve acid hydrolysis followed by derivatization, and subsequent separation and quantification using gas chromatography (GC) or HPLC. In the measurement of monosaccharide composition in PNP, Xiang Ying^61^ emphasized that complete hydrolysis of polysaccharides into monosaccharides is a critical step. This process requires strict control of acid concentration under high-temperature and strongly acidic conditions to prevent sugar carbonization. Following hydrolysis and acetylation, PNP was found to consist of three monosaccharide types, with a molar ratio of d-mannose: d-glucose: d-galactose = 3.56 : 12.2 : 1. Zhang Ping *et al.*^87^ isolated a heteropolysaccharide from *Pholiota nameko* and, through GC analysis, reported that mannose and arabinose were the most abundant monosaccharides in PNP, followed by rhamnose and xylose. Using trifluoroacetic acid (TFA) for partial acid hydrolysis and subsequent GC determination, Chen Jian^1^ identified xylose, mannose, glucose, and galactose in PNP, with a molar ratio of 1.5 : 3.36 : 14.2 : 1. Yue Qi^59^ employed TFA hydrolysis coupled with HPLC to analyze the monosaccharide composition of the PNP fraction WPNP. The results indicated molar proportions of Gal : Man : Glc : GlcA : Xyl : Me-Gal = 54.2 : 23.4 : 7.3 : 6.6 : 6.1 : 2.4. Song Miaomiao^88^ extracted five distinct polysaccharide fractions (RTSP, BWSP, HPSP, WBSP, and SASP) from *Pholiota nameko* and observed variations in their monosaccharide profiles. Mannose was the predominant monosaccharide in RTSP, BWSP, and HPSP, whereas glucose was most abundant in WBSP and SASP, although mannose also remained significantly present. With ongoing advancements in instrumentation and continuous refinement of detection protocols, more sensitive and accurate methods for monosaccharide determination have been developed. For instance, Zhao Hang^89^ utilized ion chromatography coupled with pulsed amperometric detection (IC-PAD), which offers exceptionally high sensitivity, and identified mannose, glucose, and galactose as the constituent monosaccharides. In summary, the monosaccharide composition of PNP varies with extraction methods, which may be attributed to differences in polysaccharide isolation conditions and detection methodologies. Furthermore, the proportional distribution of monosaccharides differs among various PNP fractions; however, mannose represents the most abundant monosaccharide in the majority of cases.

### 4.5 Linkage type

PNP comprises multiple homogeneous fractions, each of which exhibits diverse primary structures. These structures are formed through various glycosidic linkages arranged in distinct patterns, resulting in highly complex and chemically heterogeneous polysaccharide chains. Common analytical techniques for elucidating polysaccharide linkage patterns include periodate oxidation, Smith degradation, methylation analysis, nuclear magnetic resonance (NMR) spectroscopy, Congo red assay, and β-elimination reactions.^90^ Integrating multiple complementary methods can significantly enhance the accuracy and reliability of structural characterization.

Zhang *et al.*^91^ isolated a novel polysaccharide, designated SPN, from *Pholiota nameko* using high-temperature and high-pressure extraction. Following purification by column chromatography and phosphorylation modification, a purified fraction named PPN was obtained. Methylation analysis revealed that the backbones of both SPN and PPN are composed of the following glycosidic linkages: → 1,4)-glucopyranose (Glcp) → 1,6)-galactopyranose (Galp), → 1,2)-rhamnopyranose (Rhap), and → 1,6)-mannopyranose (Manp), with terminal Glcp and Arabinofuranose (Araf) residues. The side chain of SPN was identified to include → 1,4,6)-Galp and → 1,2,5)-Araf, while that of PPN consists of → 1,4,6)-Galp and → 1,2,4)-Glcp.

Sovrani *et al.*^62^ isolated a polysaccharide fraction, designated bG-PN, from *Pholiota nameko* using cold water extraction and subsequent purification. Methylation analysis and NMR spectroscopy revealed that the backbone of bG-PN contains → 3)-Glcp-(1 → linkages, with side chains attached at the O-6 position consisting of → 6)-Glcp-(1 → units. These results indicate that bG-PN features a (1 → 3)-linked backbone and branched side-chain structures. Similarly, Abreu *et al.*^60^ purified a β-d-glucan from PNP. Through methylation analysis and NMR characterization, they confirmed the presence of a (1 → 3)-linked backbone, with side branches likely composed of multiple Glcp units connected *via* (1 → 6) linkages. The number of Glcp units in the side chains was suggested to influence the gel strength of the β-d-glucan at varying temperatures.

Xiong *et al.*^16^ purified two structurally distinct fractions, BWSP-1 and BWSP-2 methylation analysis indicated that both BWSP-1 and BWSP-2 contain four types of sugar residues within their backbones. ^1^H NMR analysis of BWSP-1 revealed the presence of two anomeric configurations (α and β), corresponding to the glycosidic linkages: → 6)-β-d-Manp-(1 →, → 3,6)-β-d-Manp-(1 →, and → 4)-α-d-Glcp-(1 →. Additionally, the O-3 position of → 6)-β-d-Manp-(1 → was identified as the attachment site for a side chain consisting of β-d-Xylp-(1 →. The backbone of BWSP-2 was determined to comprise → 3,6)-β-d-Manp-(1 →, →6)-d-Manp-(1 →, and → 4)-β-d-Manp-(1 →, with a side chain of → 4)-β-d-Xylp-(1 → linked at the O-3 position of → 3,6)-β-d-Manp-(1 →.

Yue *et al.* obtained the WPNP from fresh *Pholiota nameko via* hot-water extraction, separation and purification were performed using DEAE-cellulose ion-exchange chromatography and Sepharose CL-6B, yielding two polysaccharide fractions named WPNP-N-b and WPNP-A-a.^59^ Based on methylation analysis and NMR spectroscopy, the backbone of WPNP-N-b was determined to consist of → 6)-α-d-Galp-(1 → and → 2,6)-α-d-Galp-(1 →, with the O-2 position of certain Galp residues substituted by terminal β-d-Manp. Meanwhile, the backbone of WPNP-A-a was identified as → 3)-β-d-Manp-(1 →, with terminal α-d-Xylp attached at the O-2 and O-6 positions. Additionally, d-glucuronic acid (GlcA) was suggested to be present in side chain structures.

Chen *et al.* isolated a PNP with a narrow molecular weight distribution using water extraction followed by ethanol precipitation.^92^ Structural analysis through periodate oxidation, Smith degradation, and methylation analysis indicated that PNP contains glycosidic linkages of the types 1 → 2, 1 → 3, and 1 → 6, corresponding to C–C single bonds. ^1^HNMR spectroscopy revealed the presence of both α and β glycosidic configurations, with the β form being predominant. In the β-elimination reaction, one group of PNP was treated with 0.2 mol L^−1^ NaOH in a water bath for 1.5 h, while another group remained untreated. The alkali-treated O-glycosidic bonds generated α-aminocrotonic acid and α-6-aminobutenoic acid. Ultraviolet spectroscopy showed an increased absorbance at 240 nm for the alkali-treated sample compared to the untreated control, confirming the presence of O-glycosidic bonds in PNP. Furthermore, the iodine-potassium iodide test suggested that PNP may possess extended side chains and a highly branched architecture.

The differences in the glycosidic linkage patterns of PNP may be attributed to variations in extraction methods. Zhang *et al.* employed a high-temperature and high-pressure extraction approach, which tends to solubilize complex heteropolysaccharides tightly bound to proteins, cellulose, and other structural components.^91^ In contrast, Sovrani *et al.* used a cold-water extraction method, which may better preserve heat-sensitive or cold-water-soluble β-glucans, thereby influencing the glycosidic bond composition in the extracted PNP.^62^ Xiong *et al.* utilized multiple extraction techniques, including ambient-temperature water extraction, alkali extraction, and high-pressure extraction, to repeatedly isolate polysaccharide fractions with diverse linkage patterns.^16^ Furthermore, the *Pholiota nameko* samples used in these studies were sourced from different geographical origins, suggesting potential variations in fungal strains, developmental stages, and growth conditions. Overall, biological and environmental differences likely contribute to the observed discrepancies in glycosidic linkage profiles among extracted PNPs.

### 4.6 Microstructure

Cryogenic electron microscopy (cryo-EM) serves as a pivotal technique for microscopic investigation and has greatly advanced the field of structural biology.^93^ When the mass concentration of PNP reached 0.6 g mL^−1^, the internal structure of the PNP-chitosan (PNP-CS) composite system displayed a uniform honeycomb-like architecture with regular pores. However, at PNP concentrations exceeding 0.6 g mL^−1^, the pore structure became irregular, the integrity of the matrix was compromised, and cracking occurred, suggesting that pore morphology is influenced by intermolecular interactions.^63^ Scanning electron microscopy (SEM) is widely used to characterize the morphological features of polysaccharides.^32^ Xiong Shanqiang *et al.*^16^ extracted PNP (BWSP) *via* hot water extraction and observed an irregular flake-like morphology at 500× magnification. At 100 00× magnification, BWSP-1 exhibited a granular and rough surface, whereas BWSP-2 maintained a smooth and continuous sheet-like structure, indicating relatively strong repulsive and weak attractive forces among PNP molecules. Zhang Xu *et al.*^47^ observed through electron microscopy that the PPN structure is compact with some lamellar structures on the surface, while the CPPN structure is loose with irregular plate-like structures. Su Yuming *et al.*^94^ conducted SEM imaging of PNP and reported an irregular, flake-like overall morphology with a dense and smooth surface, suggesting that PNP may exhibit favorable stability and bioactivity. Atomic force microscopy (AFM), developed over the past decade, is a valuable tool for analyzing the morphology and conformation of biological macromolecules such as polysaccharides.^95^ AFM observations revealed that PNP molecules were distributed in a nearly linear arrangement on mica substrates without aggregation, which is attributed to intermolecular repulsive forces dominating over attractive interactions.^26^ Anggara K *et al.* combined electrospray deposition with scanning tunneling microscopy (STM), enabling direct visualization of the primary structures of complex glycoconjugates—including multiply glycosylated glycoproteins—as well as individual amino acids and monosaccharides within recognition molecules. This approach allows precise identification of specific proteins/glycoproteins within complex biological mixtures.^96^ It is reported that the higher-order structure of polysaccharides exerts a more profound influence on their functional properties than their primary structure. However, due to the structural complexity and low crystallinity of polysaccharides, studying their higher-order architecture remains challenging. The advent of AFM and STM has opened new avenues for such investigations: these techniques can produce stable and high-resolution images of higher-order polysaccharide structures, providing powerful tools for in-depth structural studies and showing broad application potential.^97^

Regarding the structural features of PNP, Xiang Ying *et al.*^1^ identified it as a mucopolysaccharide characterized by a complex polysaccharide backbone conjugated with proteins. PNP exhibits high content of uronic acid and amino sugars, along with a diverse profile of conjugated amino acids, reaching a total content of 0.714%. Han Dan *et al.*^58^ further revealed that PNP is primarily composed of glucose and mannose at a molar ratio of 4.24 : 1.00. Its backbone consists of (1 → 3)-linked β-d-Glcp and (1 → 3)-linked β-d-Manp, while side chains are comprised of (1 → 3,6)-linked Glcp, (1 → 3,6)-linked Manp, terminal Glcp, and polyphenols. Recent studies indicate that PNP is a glycoconjugate with a triple-helix conformation, extended side chains, and a high degree of branching, which provides a theoretical foundation for its application development. However, detailed structural aspects—such as monosaccharide positioning and sequence, ring size, and specific linkage patterns—remain to be fully elucidated. Current structural research on polysaccharides predominantly focuses on primary structures. Studies concerning the purification of PNP, the biological activities of purified fractions, and comprehensive structural characterization remain relatively limited. In this study, a detailed investigation into the primary structure of PNP was conducted.^58^ Therefore, future work should integrate classical chemical methods with advanced instrumental techniques to further explore the fine structural features and physicochemical properties of PNP.^98^

## 5. Bioactivities of PNP

**Fig. 3:**
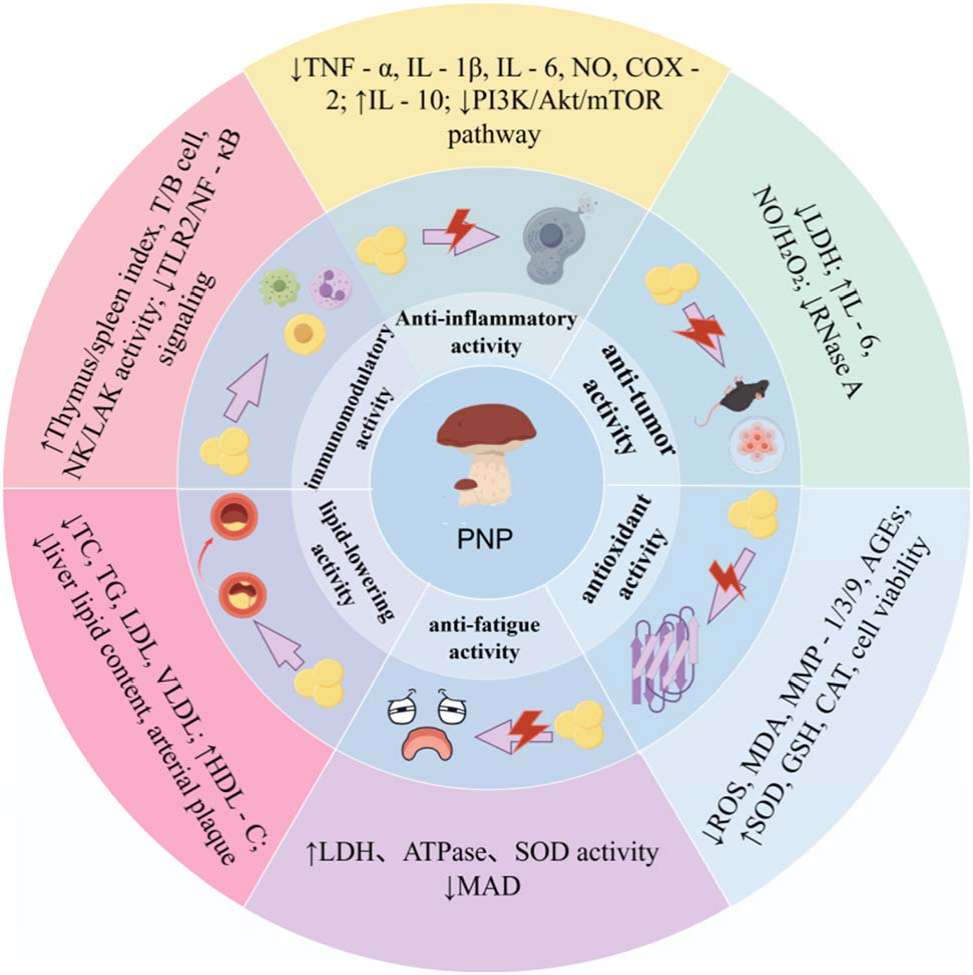
Biological activity and mechanism of PNP.

PNP exhibits a wide range of notable biological activities, demonstrating considerable potential in various fields including antioxidant, anti-aging, immunomodulatory, anti-tumor, anti-inflammatory, and hypolipidemic applications. As summarized in Fig. 3, the figure presents an overview of the relevant bioactivities and mechanisms of action associated with PNP. Its bioactivities are manifested not only in fundamental functions such as scavenging free radicals, protecting cells, and regulating immune cells and cytokines, but can also be further enhanced through structural modifications—for instance, phosphorylation or iron chelation. Moreover, the efficacy of PNP has been confirmed through both *in vitro* experiments and *in vivo* animal models, thereby providing a solid foundation for its further development and application in medicinal and health-related products.

### 5.1 Antioxidant activity

Antioxidant activity helps combat oxidative stress and protect cellular components through multiple mechanisms.^99^ This process relies on an integrated triad mechanism involving the endogenous defense system, exogenous intake, and health regulation.^100^ Exogenous polysaccharides from edible mushrooms significantly enhance this system by boosting antioxidant enzyme activity, inhibiting lipid peroxidation, and scavenging free radicals such as hydroxyl radicals.^66^ As a notable example, PNP exhibits strong antioxidant properties, supported by consistent evidence from both *in vitro* and *in vivo* studies.^101^ In evaluating PNP's bioactivity, You *et al.*^19^ employed a multi-dimensional approach combining chemical analysis, cell experiments, and mouse models. Treatment with 0.5 mg mL^−1^ PNP in cultured cells showed protective effects comparable to the positive control BHT, effectively maintaining redox homeostasis. A reducing power assay (Prussian blue method) yielded an absorbance value (*A*_700_) of 0.1, further supporting its antioxidant role. Using an H_2_O_2_-induced oxidative damage model in RAW 264.7 cells, Lan Zheng *et al.*^102^ demonstrated that sulfated degraded PNP (S-PNPS) significantly increased cell viability compared to the native polysaccharide. These cellular results aligned with chemical antioxidant assays, confirming that sulfation enhances PNP's bioactivity. Additional studies^103^ revealed varying antioxidant capacities among fractionated PNP components, with PNPS-60 showing the strongest free radical scavenging and reducing power in *in vitro* tests.

Structural modification is a key strategy for enhancing the antioxidant activity of PNP, with cell-based assays providing critical evidence for the efficacy of modified derivatives. Iron-chelated derivatives CP-1-Fe and CP-3-Fe,^104^ derived from polysaccharides isolated of *Lepista sordida* and *Pholiota nameko* co-culture, demonstrated enhanced protective effects in oxidative stress-induced hepatocyte injury. Compared to the unmodified forms, cells treated with CP-1-Fe and CP-3-Fe showed a 20–30% decrease in malondialdehyde and a 15–25% increase superoxide dismutase activity. Although minor variations were observed in certain chemical antioxidant assays, the cellular protective effects were significantly stronger. Moreover, the iron in these complexes was effectively absorbed by cells, suggesting dual antioxidant and iron-supplementing potential.^105^ Similarly, phosphorylation markedly improved the antioxidant performance of PNP. In H_2_O_2_-injured human umbilical vein endothelial cells, treatment with 3 mg mL^−1^ phosphorylated PNP (PPN) increased cell viability to 82.3%, significantly higher than the 61.5% with unmodified PNP (SPN) at the same concentration. PPN also reduced intracellular reactive oxygen species (ROS) levels by over 35% compared to the SPN group, as measured by fluorescence probing, with effects showing clear concentration dependence.^47^ These findings indicate that phosphorylation enhances the cellular antioxidant efficacy of PNP by improving polysaccharide-cell interactions, supporting its potential application in cytoprotection.

### 5.2 Anti-aging

**Fig. 4:**
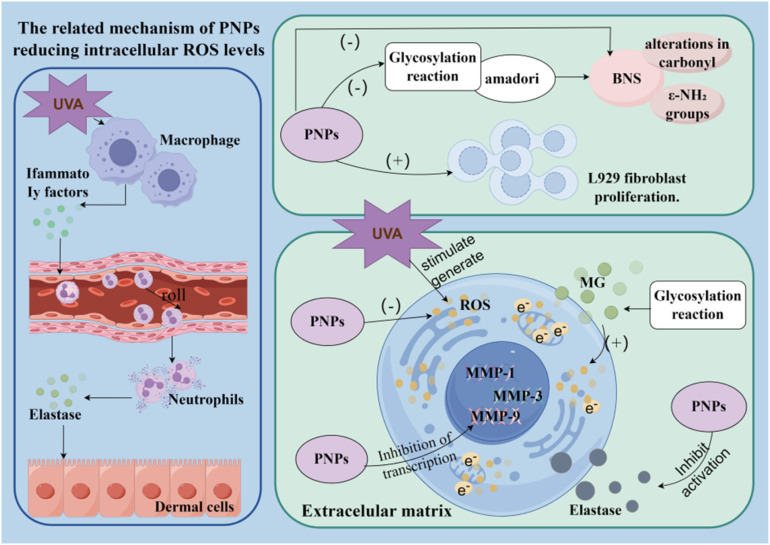
The anti-aging mechanism of PNP on skin.

PNPs demonstrate considerable potential in skin anti-aging and repair. Glycation is a non-enzymatic reaction that ultimately leads to the formation of structurally stable compounds termed advanced glycation end products (AGEs). Excessive accumulation of AGEs exacerbates the degradation of physiological functions and accelerates aging. Skin elastin, collagen, and the specific receptor RAGE (receptor for advanced glycation end products) are particularly susceptible to reaction with AGEs, resulting in functional impairment. Studies indicate that PNPs can significantly suppress the formation of Amadori products, reduce methylglyoxal (MG)-induced alterations in carbonyl and ε-NH_2_ groups in bovine serum albumin (BSA), and inhibit both the aggregation of glycated proteins and the intensity of associated fluorescent signals, as evidenced by sodium dodecyl sulfate-polyacrylamide gel electrophoresis (SDS-PAGE) and fluorescence analyses.^106^ Among the various fractions, PNP-80 exhibited superior anti-glycation efficacy compared to PNP-40 and PNP-60. Furthermore, PNPs markedly increased the survival and growth rates of Hs68 cells under MG-induced stress while reducing intracellular ROS levels. PNP-80 showed the strongest protective effect at a concentration of 1.5 mg mL^−1^.^106^ These findings suggest that PNPs possess notable antioxidant and ROS-scavenging capacities, thereby protecting cells against glycation-mediated damage. Matrix metalloproteinases (MMPs) play a critical role in extracellular matrix (ECM) degradation, and their overactivation accelerates skin photoaging. Research has shown that PNPs significantly inhibit elastase activity, attenuate UVA-induced ROS generation, and downregulate the expression of MMP-1, MMP-3, and MMP-9, indicating strong anti-photoaging potential. The underlying mechanism is illustrated in Fig. 4. Moreover, studies report that PNPs suppress elastase activity in a dose-dependent manner: at a concentration of 500 µg mL^−1^, the inhibition rates reached 43% for PNP-40, 47% for PNP-60, and 54% for PNP-80.^107^*In vitro* experiments further revealed that PNPs exhibit significant antioxidant and anti-collagenase activities, along with a pronounced ability to promote the proliferation and migration of L929 fibroblasts while reducing intracellular ROS levels. Compared to the control group, PNP-80 performed most prominently, showing a collagenase inhibition rate of 61%, a free radical scavenging rate of 53.33%, a 5.8-fold increase in cell proliferation activity, and a 362.8% enhancement in migration rate.^108^ These results underscore the potential of PNPs as functional ingredients for applications in wound healing and skin regeneration.

### 5.3 Immunomodulatory activity

**Fig. 5:**
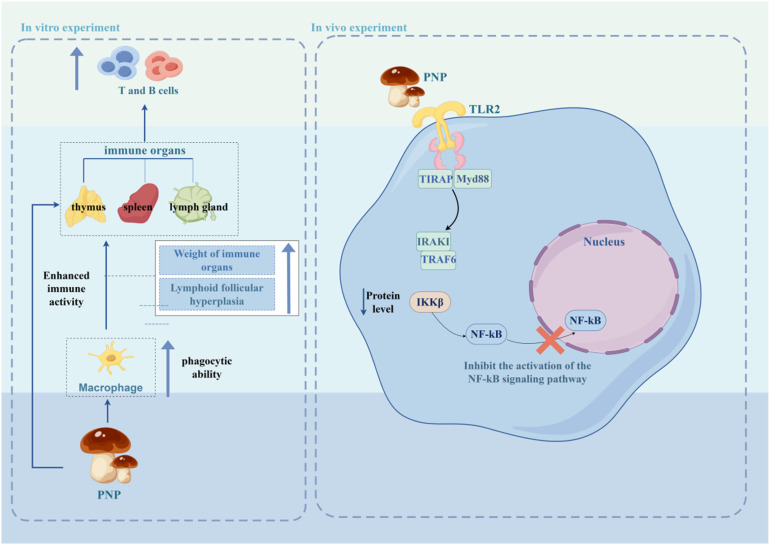
The immune regulatory mechanism of PNP.

The immune system serves as a crucial defense mechanism that protects organisms against pathogens and abnormal cells while maintaining internal homeostasis.^109^ It preserves organismal health through three core immune functions.^110^ Numerous fungal and plant polysaccharides have been shown to exhibit potent immunomodulatory activities. Structural analyses reveal that PNP shares key structural features with these bioactive polysaccharides, suggesting that PNP may also possess immunoregulatory properties.^99^ Furthermore, PNP can bolster immune function through mechanisms such as immune cell activation, regulation of cytokine secretion, and enhancement of mucosal immunity. Cui Yingjun *et al.*^5^ investigated the effects of PNP on the thymus index, spleen index, and splenic lymphocyte transformation rate in mice. Their results demonstrated that PNP enhances immune function by significantly increasing these parameters.^100^ Similarly, Lin Lili's study^101^ indicated that PNP elevates immune organ indices in mice, thereby promoting overall immune activity, though it did not stimulate hyperplasia of the immune organs. Notably, within the effective dosage range, low doses of PNP (100–200 mg kg^−1^) influenced the thymus index, whereas a higher dose (400 mg kg^−1^) did not elicit a significant immune response within the tested concentration range. PNP also enhances the phagocytic capacity of macrophages. As shown in the left panel of Fig. 5, enhanced phagocytic activity contributes to increased immune organ weight, promotes the proliferation of lymphoid nodules, and facilitates the generation of T and B cells, ultimately strengthening overall immunity.^1^ Many natural polysaccharides modulate immunity by engaging immune receptors such as toll-like receptors (TLRs), thereby activating key signaling molecules within immune cells. For instance, as shown in the right panel of Fig. 5, one study demonstrated that the PNP fraction PNPS-1 regulates mouse bone marrow-derived dendritic cells (BMDCs) *via* TLR2 and the downstream nuclear factor-κB (NF-κB) pathway. Specifically, PNPS-1 reduced mRNA expression levels of Myd88, TRAF6, TIRAP, IRAK1, IKBKB, NFKB1, NFKB2, and RelA in immature BMDCs (as measured by RT-PCR), decreased IKKβ and P65 protein levels (*via* western blot), and suppressed NF-κB P65 production (as assessed by ELISA). A direct interaction between PNPS-1 and TLR2 on the cell surface was also observed.^111^ Additionally, studies have shown that ultrasonic treatment reduces the binding affinity between PNPS and TLR2 under appropriate intensity conditions.^112^

### 5.4 Antitumor activity

**Fig. 6:**
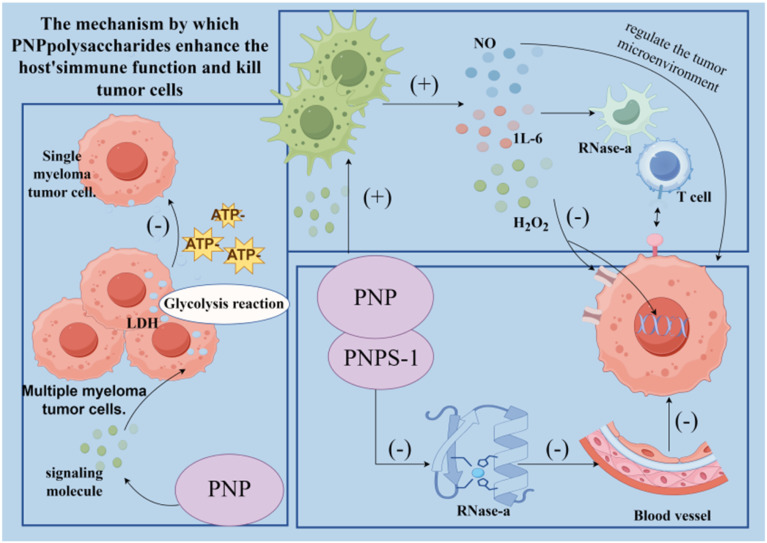
Mechanism of PNP antitumor activity.

The development of tumor cells involves multiple factors and a complex, multi-step process.^113,114^ Polysaccharides derived from various mushrooms have demonstrated significant antitumor properties,^47,115^ and numerous studies indicate that PNP also exhibits considerable tumor-suppressive effects.^1^ It has been reported that at a concentration of 0.5 mg mL^−1^, PNP achieved an inhibition rate of up to 86.6% against S-180 sarcoma cells, increased monocyte proliferation, enhanced macrophage phagocytosis to 64.17%, and boosted the activity of immune cells including lymphokine-activated killer (LAK) cells and natural killer (NK) cells.^116^ In tumor tissues, the levels of various molecules, cells, and physiological indicators are often abnormally elevated, such as tumor necrosis factor-α (TNF-α) and interleukin-6 (IL-6).^117^ In experiments conducted by Song Yuguang *et al.*,^118^ radioimmunoassay measurements revealed that the number of splenic nodules and macrophages in PNP-treated groups was consistently higher than in the blank group, with the therapeutic group also showing elevated counts compared to controls. These results suggest that PNP effectively promotes splenic proliferation and differentiation in both tumor-bearing and normal mice. Additionally, PNP can inhibit lactate dehydrogenase (LDH) activity, thereby obstructing the glycolytic pathway in myeloma tissues and suppressing further tumor growth. This mechanism is illustrated in the left panel of Fig. 6. Polysaccharides exert antitumor effects mainly through two mechanisms: direct cytotoxic activity against cancer cells and indirect immunomodulatory effects that enhance host immune function. Notably, PNP primarily acts through the indirect pathway.^1^ A series of experimental studies have confirmed the favorable antitumor activity of PNP, showing strong inhibitory effects on human prostate cancer cells (22Rv.1) and human hepatoma cells (Hep2B) in a concentration-dependent manner. At a concentration of 2.0 mg mL^−1^, the inhibition rates reached 73.51% for 22Rv.1 cells and 67.81% for Hep2B cells.^1^ Xiang Ying^1^ further investigated the antitumor activity of PNP using *in vitro* models including mouse peritoneal macrophages (RAW264.7), human prostate cancer 22Rv.1, and human hepatoma Hep2B cells. The results indicated that PNP promotes macrophage proliferation, enhances phagocytic capacity, and stimulates the secretion of nitric oxide (NO), hydrogen peroxide (H_2_O_2_), and IL-6. Moreover, PNP strongly inhibited the proliferation of both tumor cell types, with effects clearly dependent on concentration. Anti-angiogenesis may represent another pathway through which polysaccharide peptides mediate antitumor activity. Certain antitumor polysaccharides function by inactivating excessive ribonucleases in tumor tissues. For instance, as shown in the right panel of Fig. 6, studies show that PNPS-1, a fraction of PNP, inhibits bovine pancreatic ribonuclease (RNase A). Since inhibitors of RNase A may also serve as effective inhibitors of angiogenesis, this mechanism could contribute to the antitumor activity of PNP.^75^

### 5.5 Anti-inflammatory activity

**Fig. 7:**
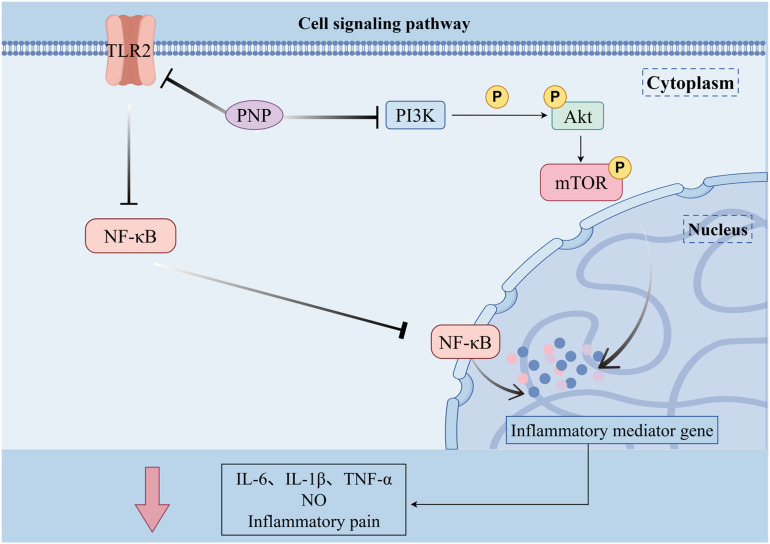
The anti-inflammatory mechanism of PNP.

PNP demonstrates significant anti-inflammatory activity (Fig. 7). In the formalin test used to evaluate the antinociceptive effect of GHW-PN, purified β-d-glucan from hot water extraction of *Pholiota nameko*, this fraction did not reduce nociception during the first phase (neurogenic pain). However, it significantly inhibited nociceptive behavior during the second phase (inflammatory pain), indicating its efficacy in alleviating inflammatory pain within this model. These results suggest that the antinociceptive effect of GHW-PN may be associated with the inhibition of inflammatory mediator synthesis or release, potentially mediated through promoting the downregulation of the NF-κB signaling pathway *via* TLR2 receptors.^60^ Studies have shown that the PNP fraction PNPS-1 can inhibit local ear edema and paw edema in mice, suppress the formation of subcutaneous cotton pellet-induced granuloma tissue in rats, and reduce peritoneal leukocyte adhesion *in vitro*. Furthermore, acute and chronic administration of PNPS-1 did not induce gastric lesions in rats, supporting its potential as an anti-inflammatory agent for the treatment of inflammation-related diseases.^119^ Both native SPN and PPN significantly reduce NO levels and progressively decrease the secretion of pro-inflammatory cytokines—including TNF-α, IL-1β, and IL-6—in a concentration-dependent manner. Among these, PPN exhibited more potent anti-inflammatory activity than SPN. Specifically, PPN dose-dependently inhibited the mRNA and protein expression of PI3K, Akt, and mTOR, thereby blocking the PI3K/Akt signaling pathway. This led to a reduction in phosphorylated mTOR (*p*-mTOR) protein levels and decreased TNF-α, IL-1β, and IL-6 secretion, ultimately contributing to its anti-inflammatory effects.^47^ Additionally, in the formaldehyde-induced inflammatory pain model, the PNP fraction GHW-PN displayed notable anti-inflammatory and analgesic activities, outperforming other previously studied β-d-glucans. However, it showed no significant effect during the neurogenic pain phase.^60^

### 5.6 Hypolipidemic effects

**Fig. 8:**
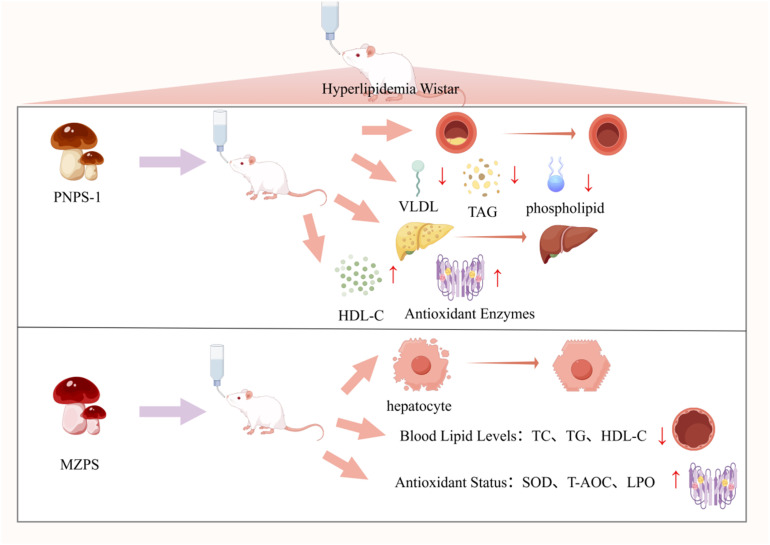
The lipid-lowering effect of PNP.

In terms of hypolipidemic effects, studies have shown that PNPS-1, a fraction derived from PNP, exerts anti-hyperlipidemic activity in hyperlipidemic Wistar rats. As shown in the upper panel of Fig. 8, its effects are manifested through multiple mechanisms: reduction of lipid components including very low-density lipoprotein (VLDL), low-density lipoprotein cholesterol (LDL-C), triglycerides (TG), and phospholipids; increase in high-density lipoprotein cholesterol (HDL-C); and decrease in the atherosclerosis index. Additionally, PNPS-1 lowers the contents of total lipids, total cholesterol (TC), TG, and phospholipids in the liver; reduces malondialdehyde (MDA) levels in both serum and liver; enhances antioxidant enzyme activities; attenuates oxidative stress; decreases overall body weight and the weight of specific organs; and ameliorates pathological alterations in coronary arteries.^12^ Further studies indicated that mycelial zinc-enriched polysaccharide (MZPS) from *Pholiota nameko* SW-02 improves lipid profiles—including TC, TG, HDL-C, LDL-C, and VLDL-C—as well as hepatic lipid levels (TC and TG) in a high-fat emulsion-induced hyperlipidemic mouse model. MZPS also enhanced antioxidant status by increasing superoxide dismutase (SOD) activity and total antioxidant capacity (T-AOC), while reducing lipid peroxidation (LPO). Histopathological examination of liver tissues and measurements of alanine transaminase (ALT) activity further revealed that MZPS alleviates hepatocyte damage.^120^ As summarized in the lower panel of Fig. 8, these results suggest that MZPS may serve as an effective agent for reducing blood lipids and preventing high-fat diet-induced hyperlipidemia and non-alcoholic fatty liver disease (NAFLD).

Although existing studies have demonstrated that PNPs exhibit multiple bioactive properties—such as antioxidant, anti-aging, immunomodulatory, antitumor, anti-inflammatory, and lipid-lowering effects—their mechanisms of action remain inadequately elucidated. Current evidence relies predominantly on *in vitro* and chemically-induced models, with a critical lack of *in vivo* validation, clinical assessments, and systematic investigation into structure-activity relationships. Notably, although polysaccharides are known to mediate systemic health benefits *via* modulation of the gut microbiota, the interaction between PNPs and the gut microbiome has not been empirically established. Future studies should integrate multi-omics technologies, genetically defined models, and translational medical research to clarify the mechanisms of action of PNPs and to explore their hitherto undiscovered role in the regulation of gut microbiota.

## 6. Structure–activity relationship

It is well established that the biological activities of polysaccharides, including those from PNP, are strongly influenced by structural features such as molecular weight, monosaccharide composition, types and patterns of glycosidic linkages, and three-dimensional structure of the polysaccharides.

### 6.1 Molecular weight and monosaccharide composition

Studies have indicated that the relatively high antioxidant activity observed in certain polysaccharides may be associated with their low molecular weight and high uronic acid content.^121^ Through analysis of molecular weight data from 41 antioxidant polysaccharides, Chen *et al.* concluded that the majority of polysaccharides with notable antioxidant properties possess low molecular weights (<10 kDa) compared to high molecular weight (>100 kDa).^122,123^ As reviewed by Wang *et al.*, PNP (<10 kDa) demonstrate superior antioxidant performance due to the greater accessibility of reducing groups (*e.g.*, hydroxyl and amino groups), which can readily react with free radicals and oxidants. In contrast, the compact PNP (>100 kDa) sterically hinders these functional groups, limiting their antioxidant efficacy.^124^ Chou *et al.* isolated three fractions (PNP-40, PNP-60, and PNP-80) from crude PNP, with molecular weights of 333.49 kDa, 21.57 kDa, and 4.40 kDa, respectively. Their results demonstrated that PNP-80, the fraction with the lowest molecular weight, exhibited the strongest antioxidant activity.^103^ Furthermore, studies have reported that the antioxidant activity of polysaccharides is influenced by their hydrogen-donating capacity.^125,126^ A higher degree of branching in polysaccharides facilitates the exposure of –OH groups, thereby enhancing their ability to donate hydrogen.^127^ Zhao Ting *et al.* discovered that SSPP11 has more side chains and a higher degree of branching compared to SCPP11, and therefore, SSPP11 exhibits a stronger DPPH radical scavenging ability.^127^ However, excessive branching may hinder interaction with reactive species, while insufficient branching could limit access to antioxidant sites.^128^ For example, antioxidant polysaccharides isolated from different medicinal plants were substituted with highly branched arabinogalactan side chains, leading to a decrease in antioxidant activity.^129^ Under normal conditions, the molecular weight of polysaccharides shows a positive correlation with their immunomodulatory potency.^130^ Polysaccharides with higher molecular weights (>100 kDa) tend to display more pronounced immunoregulatory effects, which can be attributed to their enhanced binding affinity to immune cells (*e.g.*, lymphocytes, dendritic cells, phagocytes) or receptors (*e.g.*, toll-like receptors, scavenger receptors, complement receptors), thereby triggering relevant signaling pathways such as NF-κB and MAPK cascades.^123^ Specifically, polysaccharides with high relative molecular mass (>100 kDa) exert immunomodulatory functions mainly by increasing thymic and splenic indices, promoting splenocyte proliferation, enhancing NK cell activity, activating macrophage phagocytosis, and upregulating the expression of cytokines (*e.g.*, IL-2, IL-6, IL-8, IL-10, IL-1β, IFN-γ, TNF-α, and NO) and immunoglobulins.^123^

In cases where the influence of molecular weight is less pronounced, uronic acid content becomes another critical factor affecting the antioxidant capacity of polysaccharides.^131^ Generally, a positive correlation exists between the antioxidant activity of polysaccharides and their uronic acid content.^132^ Antioxidant performance is determined by the molar ratio of monosaccharides in the composition. Higher contents of arabinose and uronic acid, coupled with a lower glucose content, are conducive to the antioxidant activity of polysaccharides from *Boletus latisporus*.^133^ During the purification of MZPS, Zheng *et al.* obtained three eluted fractions—MZPS-1, MZPS-2, and MZPS-3. Among these, MZPS-2 displayed the highest antioxidant capacity, which may be attributed to its elevated uronic acid content, lower proportion of glucose in its monosaccharide composition, and relatively low molecular weight.^121^ Similarly, Sun *et al.* reported that higher glucuronic acid content coupled with lower molecular weight can enhance antioxidant activity.^134^ To further elucidate the role of uronic acids, Wang *et al.* evaluated glucuronic acid, galacturonic acid, and polygalacturonic acid using β-carotene-linoleate emulsion, DPPH radical scavenging, and FRAP assays. The results revealed that all three uronic acids possess strong antioxidant effects, with activity descending in the order: polygalacturonic acid > glucuronic acid > galacturonic acid.^131^ Therefore, molecular weight, monosaccharide composition, and the ratio of constituent monosaccharides play crucial roles in determining the biological activities of polysaccharides. Alterations in monosaccharide composition may significantly modulate their bioactivity.^47^ Yam-derived polysaccharides rich in xylose and arabinose exhibit satisfactory antidiabetic effects.^135^ Moreover, the elevated levels of xylose and arabinose contribute to the inhibition of α-glucosidase activity, which may explain the superior bioactivity observed in polysaccharides enriched with these sugars.^136^ Structural studies on antitumor polysaccharides have revealed that bioactive polysaccharides derived from bacterial sources are predominantly composed of glucose. The presence of β-(1 → 3) glycosidic linkages in the main chain and β-(1 → 6) glycosidic bonds in the side chains has been identified as essential for their antitumor activity. Notably, modified β-(1 → 3)-d-glucans incorporating branches of d-arabinose and d-mannose exhibit potent antitumor effects.^21^ By elucidating the SAR of PNP, the structural foundation of their bioactivities can be understood, thereby guiding rational structure-based optimization and advancing the development of novel therapeutic agents.

### 6.2 Glycosidic linkage pattern

The backbone of PNP consists primarily of glucose residues interconnected by β-(1 → 3) glycosidic bonds, forming a linear structural framework.^26^ It has been reported in the literature that for lentinan exhibiting antitumor activity, if its (1 → 6) branches are removed *via* hydrolysis, the tumor-inhibitory activity will be lost.^137^ This indicates that the branched side chains of the polysaccharide are crucial for its activity, and consequently, the degree of branching naturally becomes an important indicator for measuring polysaccharide activity. For instance, NMR analysis of the purified fraction wPNP-a1 (ref. 29) indicated that its sugar rings exist predominantly in the β-pyranose form. This β-(1 → 3) linkage is a characteristic structural motif commonly found in fungal polysaccharides,^138^ and shows high similarity to those present in lentinan^139^ and *Ganoderma lucidum* polysaccharide.^140^ Side chains are attached to the main backbone *via* β-(1 → 6) glycosidic linkages, resulting in comb-like or dendritic branching patterns.^47^ Methylation analysis of PNP^47^ revealed that β-(1 → 3) bonds constitute approximately 83% of the total linkages, while β-(1 → 6) bonds account for about 17%. The degree of branching (DB) value is approximately 0.17, which falls within the optimal activity range (DB 0.20–0.33) for polysaccharides with significant immunomodulatory activity.^141^ This suggests that an appropriate degree of branching is crucial for maintaining bioactivity. Meanwhile, the side chains are generally short, typically comprising one to two glucose residues, leading to a comb-like branched architecture. This structural feature contributes to considerable steric hindrance, which may be correlated with the immunomodulatory functions of PNP.^62^ E. Kishida^142^ conducted experiments in which the antitumor activities of the four polysaccharide fractions against Sarcoma 180 solid tumor were compared. The results supported the conclusion that the antitumor-active substance is most likely the (1 → 3)-β-d-glucan rather than the protein moiety. Studies have indicated^143^ that the immunomodulatory activities of both lentinan and PNP depend on the branching density of their β-(1 → 6) side chains, with an optimal DB ranging from 0.17 to 0.33. The β-(1 → 6) branches in these polysaccharides are suggested to stabilize the triple-helical conformation by increasing molecular volume (*e.g.*, lending a chain width of 23 nm for PNP compared to 20 nm for lentinan), thereby enhancing binding specificity to the Dectin-1 receptor. Studies have demonstrated that the immune recognition of β-glucans primarily relies on the pattern recognition receptor Dectin-1.^144^ Research indicates that the branched side-chain structure of polysaccharides (*i.e.*, the degree of branching, DB) can significantly influence their binding affinity to Dectin-1.^145^ Polysaccharides with an appropriate degree of branching can more effectively cross-link multiple Dectin-1 receptors through multivalent interactions, promoting their aggregation on the cell membrane to form a ‘phagocytic synapse’, thereby potently initiating downstream immune signaling pathways.^146^ Consequently, the DB emerges as a key structural parameter determining the immunomodulatory activity of polysaccharides by regulating the efficiency and specificity of receptor recognition. Additionally, the β-(1 → 3) backbones and β-(1 → 6) side chains in both *Ganoderma lucidum* polysaccharide and PNP help stabilize the triple-helix through hydrogen bonding and steric effects. Their branching density (DB = 0.17–0.33) exhibits a positive correlation with immunomodulatory potency. Methylation analyses of both polysaccharides further confirmed that the ratio of β-(1 → 3) to β-(1 → 6) linkages is approximately 5 : 1, underscoring their high structural consistency. The antitumor activity of *Ganoderma lucidum* polysaccharide is closely associated with its branching degree (0.2–0.33) along the β-(1 → 3)-glucan backbone; excessive branching has been found to diminish bioactivity.^147^ This is because steric hindrance disrupts the helical regularity, thereby leading to reduced activity.^148^ Similarly, β-glucans in PNP with analogous structures have been shown^149^ to inhibit the PI3K/AKT/mTOR and MAPK signaling pathways, reduce HIF-1α-mediated VEGF transcription, suppress proliferation and migration of vascular endothelial cells, inhibit tumor angiogenesis, and ultimately induce apoptosis in cancer cells.

### 6.3 Three-dimensional structure

The triple-helix structure of PNP is one of the reasons for their biological activity. Studies have shown that the triple-helix conformation can enhance the binding of polysaccharides to immune cells, thereby improving tumor clearance capacity and strengthening antitumor activity.^150^ Through experimental investigations, Falch *et al.*^151^ found that (1 → 3)-β-d-glucans with a triple-helix structure and an average molar mass of less than 50 × 10^4^ g mol^−1^ or greater than 110 × 10^4^ g mol^−1^ were more effective in stimulating monocytes to secrete TNF-α. This indicates that the triple-helix structure is related to immune activation, though it may not be the sole influencing factor, as molecular weight and glycosidic linkage patterns also play important roles. Additionally, some triple-helical polysaccharides exhibit antioxidant activity, which has been attributed to this structural conformation.^152^ Zhu *et al.*^13^ purified a polysaccharide named PNP from *Pholiota nameko* fruiting bodies, and Congo red testing confirmed the presence of a triple-helix structure. PNP had a molecular weight of 1.89 × 10^6^ Da, and methylation analysis indicated that its backbone consisted of (1 → 3)-linked-Glc and (1 → 3)-linked-Man. *In vitro* antioxidant assays demonstrated that PNP possessed significant hydroxyl radical-scavenging ability. Zheng *et al.*^153^ degraded AIPS-1 with sulfuric acid at different concentrations to obtain three degraded products: AIPS-2, AIPS-3, and AIPS-4. Congo red tests showed that these degraded polysaccharides still retained the triple-helix structure. Antioxidant activity assays revealed that with increasing degradation, the antioxidant activity, reducing power, and free radical-scavenging activity of the degradation products were enhanced. The preserved triple-helix structure after sulfation, the reduced molecular weight, and the increased uronic acid content were identified as contributing factors to the improved antioxidant activity.

Chemical modifications that alter the spatial structure of polysaccharides can also affect their biological activity levels. Zhang *et al.* phosphorylated *Pholiota nameko* polysaccharide (SPN) to obtain PPN and observed changes in its spatial conformation. Methylation analysis showed that phosphorylation altered the branching structure of SPN, while Fourier transform infrared (FT-IR) spectroscopy indicated partial substitution of hydroxyl groups by phosphate groups, weakening intermolecular hydrogen bonding. The introduced phosphate groups also increased the molecular electronegativity, thereby influencing biological activity. Experimental studies revealed that PPN exhibited higher free radical-scavenging rates and stronger inhibition of inflammatory factors compared to SPN, indicating that phosphorylation enhanced the antioxidant and anti-inflammatory activities of the polysaccharide.^91^ Yu *et al.*^105^ investigated iron-chelated polysaccharides from co-cultured *Lepista sordida* and *Pholiota nameko*, finding that iron was bound to the polysaccharides *via* –OH and –COOH groups, forming a stable β-FeOOH structure. This modification increased the crystallinity and thermal stability of the polysaccharides. Antioxidant activity tests showed that the iron-chelated polysaccharide fractions had stronger hydroxyl and superoxide radical-scavenging abilities than their non-chelated counterparts, indicating enhanced antioxidant activity after iron chelation. Zhang *et al.*^64^ performed carboxymethylation on *Pholiota nameko* polysaccharide (CPPN) and similarly evaluated its antioxidant activity. The carboxymethylated polysaccharide neutralized reactive oxygen species such as DPPH and ABTS radicals by providing electrons or hydrogen atoms, with ABTS radical-scavenging rates comparable to those of vitamin C. This demonstrates that carboxymethylation improved the antioxidant activity of the polysaccharide.

In summary, the biological activities of PNP are closely related to their spatial structure. The triple-helix conformation serves as the structural basis for anticancer, antitumor, antioxidant, and immune-activating properties. Chemical modifications such as carboxymethylation, phosphorylation, and iron chelation introduce active functional groups and adjust the spatial conformation of polysaccharides, endowing them with stronger electron-donating and hydrogen-supplying capabilities, thereby significantly enhancing antioxidant activity. Based on these findings, the spatial conformation of purified PNP can be further modulated to obtain polysaccharides with superior properties.

## 7. Applications in the food industry and related bibliometric studies

In the food industry, the application value of PNP is determined by a series of key properties. Specifically, the β-d-glucans extracted from *Pholiota nameko* exhibit shear-thinning behavior, gel-forming capabilities, and viscoelastic properties. These rheological characteristics enable their use as food thickeners and gelling agents.^60^ On the other hand, bioactivity serves as a core consideration for developing functional additives from PNP, and these biological activities are closely linked to the structural features of the polysaccharides. For example, PNP with a triple-helix conformation demonstrate strong antioxidant activity, which can help protect biomembranes and combat aging, making them suitable for elderly health products.^154^ β-Glucans with (1 → 6) and (1 → 3) linkages can alleviate inflammatory pain, a property related to their molecular weight and branching degree, thereby providing a basis for developing anti-inflammatory pain additives. Therefore, this section will first explore, from the dimensions of natural rheological modifiers and stabilizers as well as bioactive components, how the key properties of PNP support its specific applications. It will then employ bibliometric analysis to reveal the research landscape and development trends in this field.

### 7.1 Functioning as natural rheological modifiers and stabilizers

The slimy texture of *Pholiota nameko* is directly attributed to the pronounced rheological properties of its polysaccharides. PNPs exhibit notable shear-thinning behavior, concentration-dependent viscosity, and the ability to form weak gel networks, particularly in fractions with high molecular weight and β-d-glucan structures featuring (1 → 3) linkages.^60,62^

Traditional food thickeners typically employ xanthan gum, which provides strong thixotropy, or gelatin, which is heat-reversible but derived from animals. Xanthan gum is produced by bacteria of the genus *Xanthomonas* and can have a molecular weight of up to 1–3 × 10^6^ g mol^−1^. At 25 °C, a 0.5% aqueous solution of xanthan gum can exhibit an apparent viscosity as high as 10^6^ mPa s at low shear rates. However, its rheological properties are affected by temperature and ionic charge.^80^ Gelatin is generally derived from fish, as well as mammalian sources such as pigskin and cowhide. For instance, laboratory-prepared cod skin gelatin gels reported by Svetlana R. Derkach *et al.* achieved a viscosity of up to 1270 Pa. However, its properties are highly temperature-dependent, and it tends to melt at around 22 °C.^81^ Sovrani *et al.* isolated the SCW fraction from polysaccharides extracted from *Pholiota nameko*. Rheological studies showed that these polysaccharides exhibited shear-thinning behavior across all tested concentrations. However, the β-d-glucan from *Pholiota nameko* required a concentration of 2% to form a weak gel. At 30 °C and a frequency of 1 Hz, its complex viscosity was estimated to be approximately 20 000 mPa s. Although the viscosity of PNP does not show a comparative advantage, the PNP fraction demonstrates excellent thermal stability. Sovrani *et al.* heated SCW polysaccharides from 5 °C to 65 °C and then cooled them back to 5 °C, measuring no significant changes in the viscoelastic modulus.^83^ Similarly, Bao^82^*et al.* subjected extracted PNP to temperature cycles from 5 °C to 80 °C and back to 5 °C, observing no notable changes in gelation behavior. This indicates that PNP possess strong thermal stability, with a network structure capable of withstanding conventional food thermal processing, offering significant advantages during pasteurization steps in food manufacturing. Furthermore, different fractions of the polysaccharide at the same concentration exhibited similar apparent viscosities, suggesting that PNP do not require complete purification prior to use, potentially reducing industrial processing costs.^83^ Additionally, these polysaccharides possess natural bioactive properties that xanthan gum and gelatin lack, such as anti-inflammatory effects. This opens up possibilities for their use as functional food additives, enhancing product texture while simultaneously boosting health value.^60^

Currently, studies have already explored the application of PNP as a food thickener or gelling agent to improve product quality, demonstrating its promising potential as a natural food additive. Liu Tingting *et al.* prepared ice cream using a mixture of 4% PNP, 0.1% CMC-Na, and 0.4% monoglyceride, and compared it with traditional ice cream containing 0.5% gelatin and 0.5% monoglyceride. The sensory quality of the PNP-enriched ice cream was comparable to that of the traditional version, while its viscosity reached 880 mPa s, slightly higher than the 870 mPa s of the conventional product. Furthermore, due to the formation of a network structure between polysaccharides and fat, which slows heat transfer, the melt resistance of the PNP ice cream improved by 2.5% compared to gelatin-based ice cream.^155^ Further research revealed that PNPs can crosslink with corn starch molecules to form a stable gel network structure, thereby significantly enhancing the viscosity of the corn starch system. As the mass concentration of PNPs increased, the setback value of the composite system exhibited a trend of first decreasing and then increasing. At a PNP concentration of 0.9 g mL^−1^, the composite system achieved its optimal performance, with the setback value reaching a minimum of 3.63 mPa s, a breakdown value of 1.77 mPa s, and a gelatinization enthalpy of 3.95 J g^−1^. These results indicate that PNPs effectively inhibit the short-term rearrangement and recrystallization of amylose, demonstrating a potent anti-aging effect. Fourier transform infrared spectroscopy further confirmed that PNPs interact with starch molecules through hydrogen bonding, with the strongest interaction observed at a concentration of 0.9 g mL^−1^. It is this strong intermolecular force that reduces the mobility of starch molecular chains, hindering their ordered rearrangement, thereby endowing native corn starch with long-term texture retention and microstructural ordering that it inherently lacks.^63^ Studies have also shown that incorporating PNPs into fermented soymilk significantly improves its physicochemical properties. As the PNP addition level increased from 0 g/100 mL to 6 g/100 mL, the viscosity of the fermented soymilk rose from 4025 ± 201 mPa s to 4785 ± 421 mPa s, significantly higher than the control group without PNP addition. Concurrently, the pH value decreased from 4.32–4.23 in the control group to 4.24–4.09 in the PNP-supplemented group, indicating a substantial increase in acidity. During 21 days of storage, the pH of the PNP-supplemented fermented soymilk remained stable without significant fluctuations, demonstrating good acidity stability. This suggests that PNPs not only enhance the viscosity and acidity of fermented soymilk but may also contribute to improved storage stability. Furthermore, the addition of PNP at concentrations of 1.2%, 1.6%, and 2.4% to fermented yogurt increased the logarithmic values of viable bacterial counts by 20.0%, 26.2%, and 32.4%, respectively, performing significantly better than yogurt supplemented with 0.12% gelatin or 0.04% pectin.^6,156^ This efficacy may stem from the ability of PNPs to interact with casein micelles and stabilize the protein-polysaccharide-water matrix. This specific interaction potential, combined with its prebiotic effect of promoting probiotic viability, creates dual functional benefits that transcend the role of a mere thickening agent. Its shear-thinning property also renders PNPs suitable for products like sauces and salad dressings, which require ideal flowability and adhesion.

### 7.2 As intrinsic bioactive components in functional products

PNPs are distinguished from inert food additives by their demonstrated diverse bioactivities, including antioxidant, immunomodulatory, and hypolipidemic effects. Thereby enabling them to function as active functional ingredients that directly contribute to the health benefits of products.

In the development of functional beverages, PNPs serve purposes beyond merely increasing viscosity or improving mouthfeel. For example, grape juice and tea beverages fortified with PNPs are specifically designed to leverage their cholesterol-binding capacity, capitalizing on this validated biological function.^157^ Similarly, incorporating PNPs into fermented soy milk enhances the survival rate of lactic acid bacteria, indicating a prebiotic-like effect that improves nutritional quality alongside physical stability.^156^ The antioxidant activity of PNPs, particularly their hydroxyl radical-scavenging ability,^58^ offers a natural approach to inhibit lipid oxidation in fat-containing foods, potentially extending shelf life while augmenting health benefits. Compared to small-molecule antioxidants such as vitamin C, PNPs may provide more sustained, system-level antioxidant effects through the modulation of cellular defense enzymes, aligning with the holistic concept of “medicinal food homology”.^76,101^ The bioactivity of PNPs can be strategically amplified. Carboxymethylated PNP (CPNP) exhibits ABTS radical-scavenging activity comparable to that of vitamin C, while phosphorylated PNP (PPNP) demonstrates superior anti-inflammatory activity relative to its native form.^39,91^ This opens avenues for developing tailored PNP derivatives for specific functional food applications—such as sports nutrition or elderly care foods—where targeted enhancement of antioxidant or anti-inflammatory support is required.

The application potential of PNPs is intrinsically linked to their unique structural characteristics. They can function not only as conventional thickeners but also as multifunctional agents for texture modification, stabilization, and health-promoting benefits, with the potential to further evolve into a platform for advanced biomaterials. Future applied research should focus on creating high-value, patentable applications that leverage these unique properties to address specific industrial challenges.

### 7.3 Related bibliometrics of PNP

**Fig. 9:**
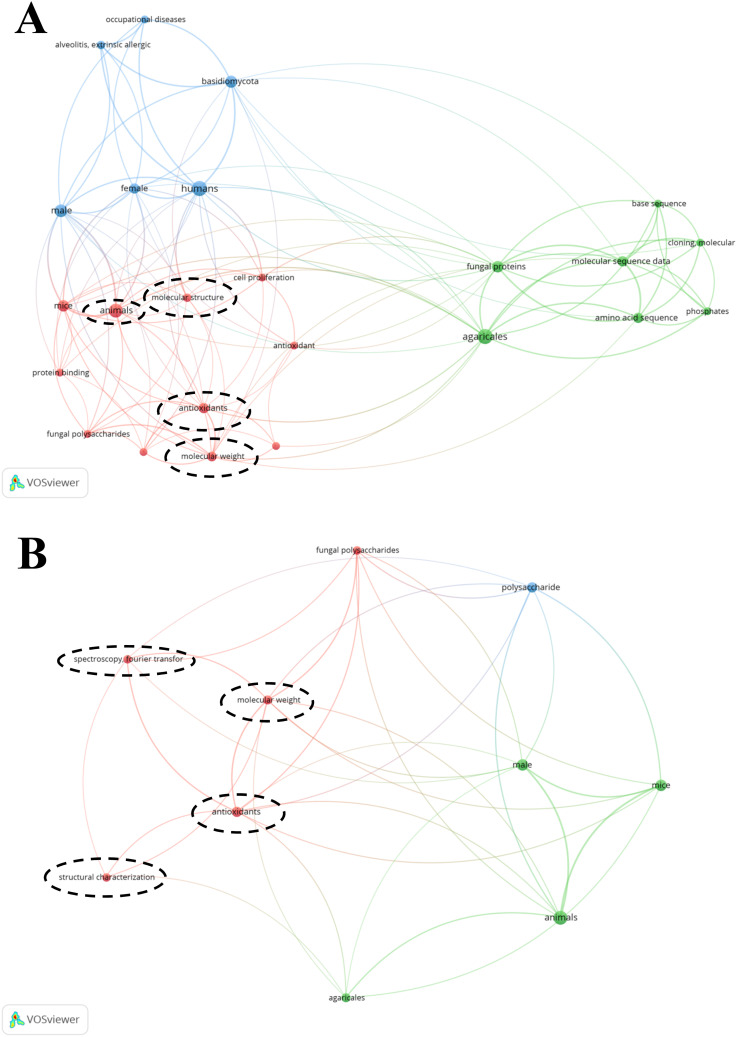
The bibliometrics of *Pholiota nameko* (A) and *Pholiota nameko* polysaccharide (B).

In the current digital era, the rapid advancement of computer science and informatics has provided strong technical support for bibliometric analysis, establishing it as a vital tool in the global scientific research landscape. Utilizing the PubMed database and keywords such as “*Pholiota nameko*” and “*Pholiota nameko* polysaccharides,” a bibliometric analysis was conducted using VOSviewer software, as shown in Fig. 9.

The results indicate that current research on *Pholiota nameko* and its polysaccharides primarily centers on four thematic areas: (1) chemical properties, including molecular weight and structural characterization; (2) biological activities, particularly antioxidant effects; (3) physiological effects in animal models such as mice; and (4) related analytical techniques, including FT-IR spectroscopy. In the domain of chemical characterization, advanced methods such as FT-IR have been widely adopted to elucidate the structural features of PNPs. With regard to biological activity, antioxidant potential has attracted substantial research attention, although its application value in antioxidation remains under continuous exploration. Animal-based studies have also investigated the bioactivity and physiological effects of these polysaccharides *in vivo*. Ultimately, the bibliometric analysis affirms the growing scientific relevance of *Pholiota nameko* and its polysaccharides within the fields of chemistry and biological sciences. Moreover, the findings highlight promising avenues for future research and the development of *Pholiota nameko*-derived functional products.

## 8. Conclusion and outlook

Edible fungi are widely recognized as “biological response modifiers” due to their dual role as natural medicinal agents and nutritional foods, capable of enhancing the body's immune defenses and sustaining core physiological vitality. In recent years, the exploration of edible fungi has emerged as a prominent research focus. Investigations into their extraction methods, structural characteristics, and physiological functions provide a solid theoretical foundation for advancing their applications in food additives and medicinal products.^158^ Among them, *Pholiota nameko* (commonly known as nameko mushroom) is extensively cultivated in Northeast and North China, and is highly favored for its fresh, slippery, tender, and crisp texture. Various extraction techniques have been developed for isolating PNP, resulting in a wide array of fractions with distinct structural features. As a result, the molecular weight of PNP spans a broad range—from several thousand to several million Daltons. Structurally, PNP is a heteropolysaccharide, predominantly composed of mannose. Its main glycosidic linkages are (1 → 6) and (1 → 3), and certain fractions exhibit branching structures and triple-helical conformations. *Pholiota nameko* exhibits a wide spectrum of biological activities, notably anti-inflammatory, antioxidant, and anti-tumor effects, highlighting its significant medicinal potential. Furthermore, the incorporation of PNP into dairy products such as yogurt has demonstrated promising results, underscoring the broad market potential of PNP as a functional food additive.^6^

Although substantial research on PNP has been conducted both domestically and internationally, numerous critical issues remain unresolved and warrant further investigation.

(1) Although various extraction methods have been developed for PNP, each approach presents specific limitations. The conventional water extraction-alcohol precipitation method is characterized by procedural simplicity and low cost, but it often results in relatively low yields. In contrast, ultrasonic-assisted and microwave-assisted extraction techniques offer higher yields and reduced processing times, making them more efficient alternatives; however, these methods frequently result in polysaccharide fractions with high levels of impurities. In recent years, more advanced techniques such as subcritical water extraction and deep eutectic solvent-based methods have been increasingly employed. These novel approaches not only improve extraction specificity and preserve the structural integrity of polysaccharides, but are also more environmentally sustainable. Concurrently, the establishment of standardized extraction protocols is crucial to ensure experimental reproducibility and cross-study comparability, thereby providing a solid foundation for in-depth investigation into the structure-activity relationships and biological functions of PNP.

(2) Despite progress in the structural elucidation of PNP, several key aspects remain unclear. The diversity of extraction and purification methods results in a wide range of PNP fractions with structurally complex and heterogeneous features. Most current studies have focused on the characterization of individual PNP fractions, lacking a holistic understanding of the overall polysaccharide composition. Furthermore, the glycosidic linkage patterns within PNP remain insufficiently characterized; existing research is largely limited to primary structural features, while higher-order structural information—closely related to PNP's biological activities—remains scarce. These gaps have hindered the elucidation of the structure-activity relationship (SAR) of PNP. To overcome these limitations, future investigations should adopt integrative analytical approaches. For example, combinations of spectroscopic techniques such as NMR and FT-IR, mass spectrometry-based methods (*e.g.*, LC-MS/MS), and advanced higher-order structural techniques—including X-ray crystallography^159^ and circular dichroism (CD) spectroscopy^160^—can provide deeper insights into both the primary and three-dimensional structures of PNP.

(3) Clinical investigations into the biological activities of PNP remain limited. Although the active components of PNP exhibit potent pharmacological effects, current research on their biological functions—such as antitumor, anti-inflammatory, and anti-aging activities—has been largely confined to preclinical studies in animal models. Reports of clinical trials involving PNP are scarce. To comprehensively elucidate the mechanism of action of PNPs at the molecular and cellular levels, integration of multi-omics technologies—such as single-cell RNA sequencing, spatial transcriptomics, proteomics, and metabolomics—offers a powerful strategy to gain deeper insights into its mode of action, including cell type-specific responses, tissue microenvironment modulation, and systemic metabolic effects. As such, the development of PNP-based therapeutic agents necessitates further clinical validation to establish safety, efficacy, and application parameters, thereby providing a solid evidence base for their medical use.

(4) Studies on the structural modification of PNP remain relatively scarce. Although various homogeneous fractions can be isolated from crude PNP, targeted structural adjustments and chemical modifications of these fractions may significantly broaden their physicochemical and biological properties. For instance, carboxymethylated PNP has been shown to possess enhanced antioxidant activity, while polyphosphorylated PNP exhibits both stronger antioxidant and anti-inflammatory effects.^64,91^ Literature demonstrates that acetylated modification of polysaccharides derived from *Pholiota adiposa* markedly enhances their immunostimulatory and anti-inflammatory activities.^161^ By analogy, acetylated PNP is anticipated to confer similar immunomodulatory properties, positioning it as a promising candidate for immunotherapeutic applications.

(5) The application of PNP as food additives has been relatively underexplored, despite their considerable market potential. As a natural compound derived from edible fungi, PNP offers excellent biocompatibility and minimal side effects, making it a promising candidate for incorporation into functional food systems. The development of PNP-based food additives could enable the production of a wide range of products with targeted health benefits and cross-industry integration. However, existing research on commercial formulations or products incorporating PNP remains limited, highlighting the need for further applied studies to support industrial translation.

In summary, this review outlines the current research progress on PNP, with a focus on extraction and purification methods, chemical structural characterization, and biological activities. It provides valuable insights for ongoing research in the PNP field and establishes a theoretical foundation for the development of PNP-based pharmaceuticals and its application in functional food products. To date, notable advancements have been achieved in the areas of PNP extraction, structure elucidation, biological activity evaluation, and preliminary food additive applications. However, several key challenges remain to be addressed. Future research should prioritize the development of efficient and environmentally friendly extraction technologies to improve yield and purity; conduct in-depth investigations of higher-order structures to clarify SAR; strengthen clinical studies to validate the therapeutic potential of PNP; expand chemical modification strategies to produce derivatives with enhanced bioactivity; and accelerate its application as a food additive to develop innovative functional food products. Through interdisciplinary collaboration, sustained exploration, and technological innovation, the potential of PNP can be further unlocked—offering new opportunities and solutions for the advancement of both the biomedicine and functional food industries.

## Author contributions

Jialu Sun: conceptualization, writing – original draft. Jiali Zhang, Yangyi Lai, and Miao Ding: methodology. Xiaoxia Kong and Mingqi Han: data curation. Zheng Li and Yifei Bian: writing – reviewing & editing, funding acquisition.

## Conflicts of interest

No primary research results, softor code have been included and no new data were generated or analysed as parof this review.

## Data Availability

No primary research results, software or code have been included and no new data were generated or analysed as part of this review.
